# Single-cell analyses reveal early thymic progenitors and pre-B cells in zebrafish

**DOI:** 10.1084/jem.20220038

**Published:** 2022-08-08

**Authors:** Sara A. Rubin, Chloé S. Baron, Cecilia Pessoa Rodrigues, Madeleine Duran, Alexandra F. Corbin, Song P. Yang, Cole Trapnell, Leonard I. Zon

**Affiliations:** 1 Stem Cell Program and Division of Hematology/Oncology, Boston Children’s Hospital and Dana-Farber Cancer Institute, Boston, MA; 2 Department of Immunology, Blavatnik Institute, Harvard Medical School, Boston, MA; 3 Stem Cell and Regenerative Biology Department, Harvard University, Cambridge, MA; 4 Department of Genome Sciences, University of Washington, Seattle, WA; 5 Howard Hughes Medical Institute, Boston Children’s Hospital, Boston, MA

## Abstract

The zebrafish has proven to be a valuable model organism for studying hematopoiesis, but relatively little is known about zebrafish immune cell development and functional diversity. Elucidating key aspects of zebrafish lymphocyte development and exploring the breadth of effector functions would provide valuable insight into the evolution of adaptive immunity. We performed single-cell RNA sequencing on ∼70,000 cells from the zebrafish marrow and thymus to establish a gene expression map of zebrafish immune cell development. We uncovered rich cellular diversity in the juvenile and adult zebrafish thymus, elucidated B- and T-cell developmental trajectories, and transcriptionally characterized subsets of hematopoietic stem and progenitor cells and early thymic progenitors. Our analysis permitted the identification of two dendritic-like cell populations and provided evidence in support of the existence of a pre-B cell state. Our results provide critical insights into the landscape of zebrafish immunology and offer a foundation for cellular and genetic studies.

## Teaser

Single-cell RNA sequencing of zebrafish hematopoiesis provides new insights into zebrafish B- and T-cell development and cellular diversity within the primary lymphoid organs (kidney marrow and thymus). This work advances the current understanding of zebrafish immunology and will facilitate future genetic and developmental studies.

## Graphical Abstract

**Figure:**
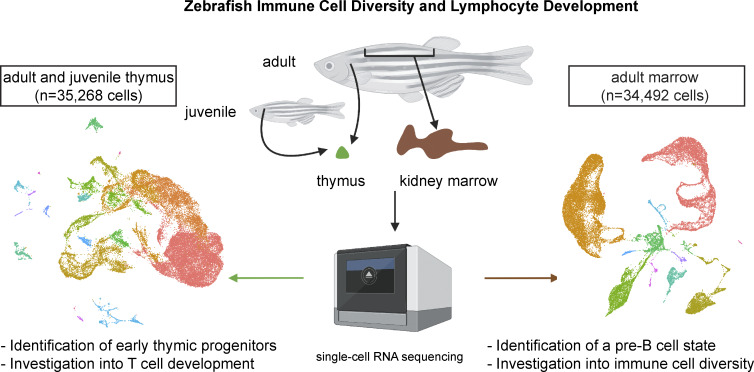

## Introduction

The zebrafish is a useful immunological model, but much remains unknown about zebrafish immune cell development (Trede et al., 2004). Although work has been done to characterize zebrafish TCR and B cell receptor loci (Haire et al., 2000; Meeker et al., 2010; Seelye et al., 2016) and track the seeding and development of the thymus (Bertrand et al., 2008; Kissa et al., 2008; Lam et al., 2002; Murayama et al., 2006; Willett et al., 1997b), literature on the intricacies of T- and B-cell development is sparse and these processes are often inferred from other teleosts (Bajoghli et al., 2019; Barraza et al., 2020; Liu et al., 2017; Page et al., 2013). Mutagenesis studies have proven fruitful for identifying conserved aspects of lymphocyte development (Schorpp et al., 2006), but functional assays in teleosts often lack specificity as few monoclonal antibodies exist for cell type purification and targeted stimulation or inhibition (Lugo-Villarino et al., 2010; Mitra et al., 2010). Creative use of existing technology in zebrafish can overcome these shortcomings to more precisely characterize immune cell populations and suggest more specific targets for downstream functional studies.

Single-cell RNA sequencing (scRNA-seq) has emerged as a powerful tool to reveal underappreciated cellular diversity and developmental trajectories. Great technological advances have enabled the capture and profiling of thousands of individual cells from varied tissues across different organisms (Svensson et al., 2018). Many computational structures and algorithms have been developed for extracting new biological insights from scRNA-seq data. This has afforded researchers opportunities to go beyond basic questions of cellular identification and tissue-specific population quantification to generate comprehensive organism-level cell atlases, discover novel subpopulations, and explore developmental processes. Trajectory-inferring algorithms, including graph-based methods like PAGA (Wolf et al., 2019), tree-based methods like Monocle (Cao et al., 2019; Qiu et al., 2017; Trapnell et al., 2014), and multifurcation methods like FateID (Herman et al., 2018), have further expanded our capability to explore questions relating to cellular ancestry. Although these methods cannot, on their own, provide definitive orderings of cellular development, their pseudotime predictions are powerful for identifying gene targets for future study. Overall, scRNA-seq provides vast opportunities to advance the field of zebrafish immunology.

Building upon the groundwork of transcriptional analyses of adult zebrafish hematopoiesis (Carmona et al., 2017; Hernandez et al., 2018; Macaulay et al., 2016; Moore et al., 2016; Tang et al., 2017), we capitalized on recent technological advances to take a deep dive into zebrafish immune cell development. We transcriptionally profiled the zebrafish kidney marrow and thymus, which uncovered new insights into zebrafish T- and B-cell development. Specifically, we observed evolutionarily conserved transcriptional regulation and developmental states, including populations consistent with early thymic progenitors (ETPs) and pre-B cells. We also identified previously uncharacterized immune cell populations in the zebrafish, including those resembling conventional type 1 dendritic cells (DCs) and plasmacytoid DCs. This knowledge builds upon our evolutionary understanding of T-cell development (Bajoghli et al., 2009), clarifies some discrepancies in the field, and better situates the zebrafish as an immunological model moving forward. Our findings reveal new genetic targets and populations prime for investigation to characterize zebrafish immunology.

## Results

### The adult zebrafish thymus exhibits rich cellular heterogeneity akin to the mammalian thymus

**Figure 1.**
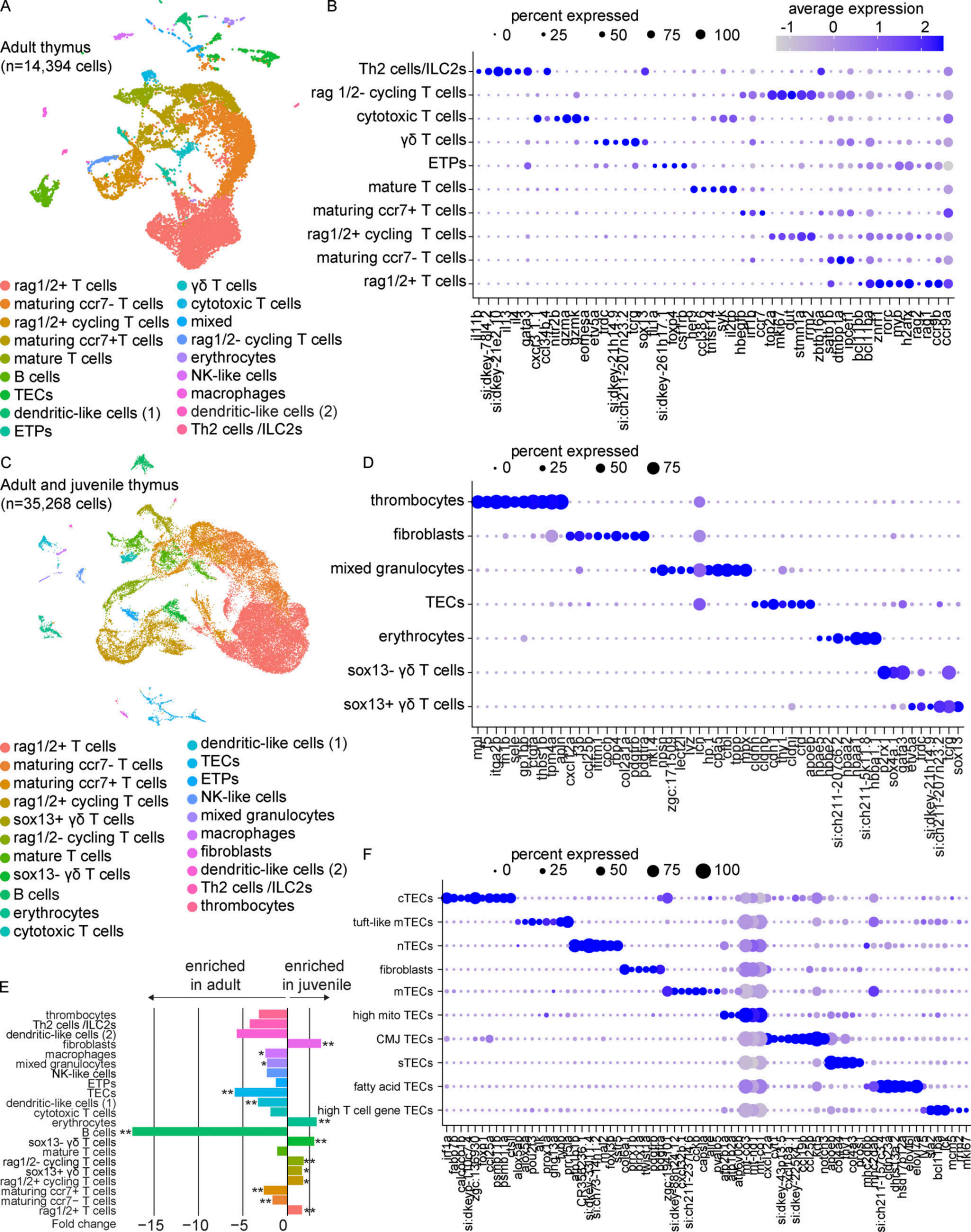
**Transcriptional characterization of the zebrafish thymus** **.** **(A)** UMAP visualization of 14,394 cells obtained from the thymi of four adult zebrafish (3–4 mpf). All thymi were dissected and processed within the same experiment. Cell type annotations were based on enriched markers (Table S1) that were identified by Wilcoxon rank sum test. **(B)** Dot plot of selected markers of the adult thymus showing expression across T cell populations. See Fig. S1 A for full dot plot. Dot size reflects the percentage of cells within a population expressing a given marker and dot color shows the average expression within that population. **(C)** UMAP visualization of 35,268 cells obtained from the thymi of four adult zebrafish and four technical replicates of juvenile zebrafish (4 wpf; pool of cells from 21 zebrafish). Cell type annotations were based on enriched markers (Table S2) that were identified by Wilcoxon rank sum test and nomenclature consistent with (A) was used when appropriate. The adult and juvenile thymi were dissected and processed in independent experiments. **(D)** Dot plot of selected markers of the adult and juvenile thymi highlighting the expression of newly/better-resolved clusters following integration. **(E)** Bar plot of the beta-binomial test comparing cell type composition of the juvenile and adult thymi (Table S3). The average fold change for each cell type is depicted by bar length and direction, with negative values enriched in the adult thymus and positive values enriched in the juvenile thymus. Statistical significance is shown for Benjamini-Hochberg adjusted P values: *, P < 0.05; **, P < 0.01. **(F)** Dot plot of selected markers distinguishing the epithelial and fibroblast-like populations from the subset analysis visualized by UMAP in Fig. S2 A and identified by the Wilcoxon rank sum test (Table S5). nTEC, neural TEC; mito, mitochondrial-gene expressing; CMJ, corticomedullary junction; sTEC, structural TEC.

**Figure S1.**
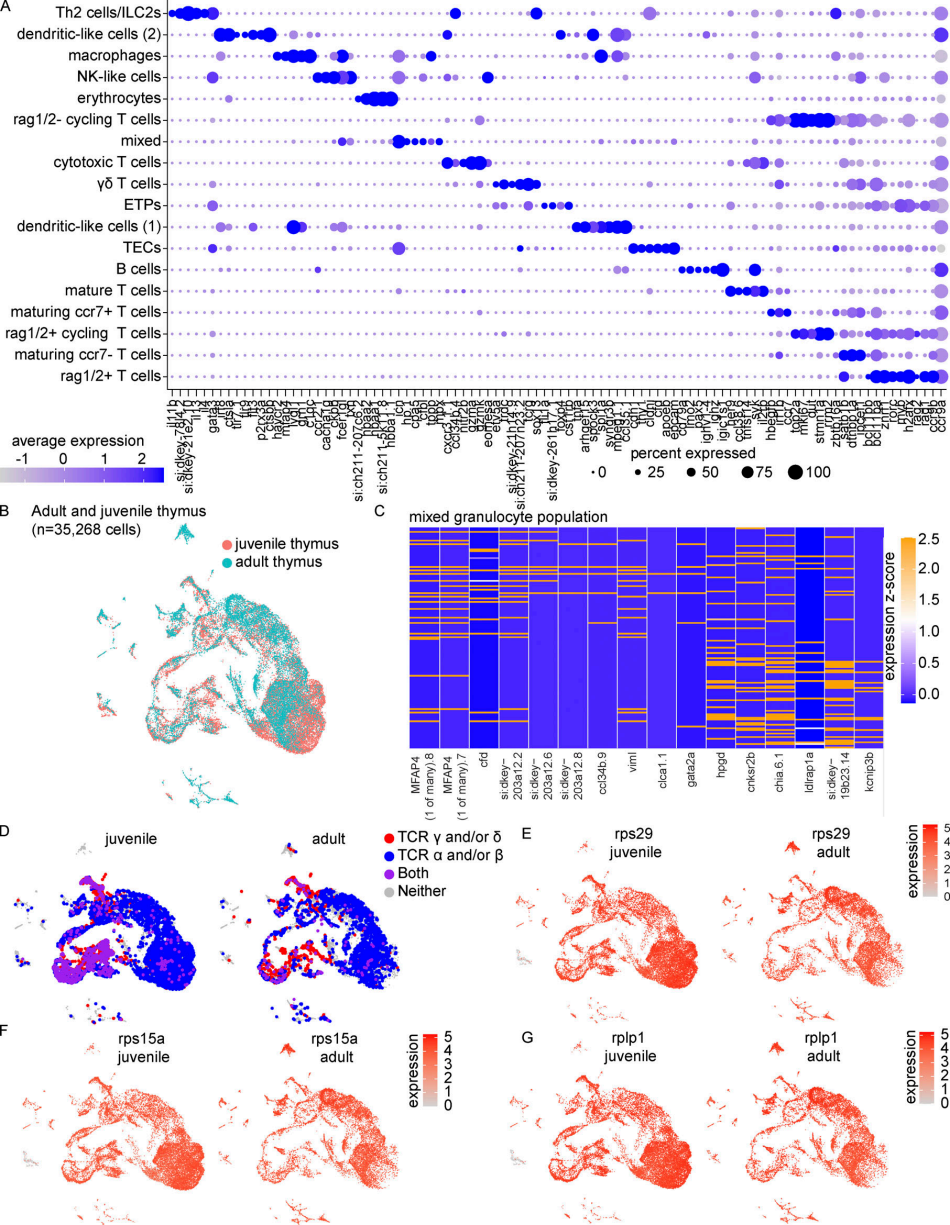
**Extended cell type characterizations of the zebrafish thymus. (A)** Dot plot of selected markers of the adult thymus showing expression across all cell populations, an expanded version of Fig. 1 B (see also Table S1). Dot size reflects the percentage of cells within a population expressing a given marker and dot color shows the average expression within that population. Similarly to Fig. 1, A and B, thymi were derived from four adult zebrafish (3–4 mpf) processed within the same experiment. **(B)** UMAP visualization by timepoint of the 35,268 adult and juvenile thymus cells integrated in Seurat. These cells were derived from the thymi of four adult zebrafish and four technical replicates of juvenile zebrafish (4 wpf; pool of cells from 21 zebrafish) processed in two independent experiments as in Fig. 1 C. **(C)** Heatmap visualization of selected genes to demonstrate heterogeneity within the mixed granulocyte population as determined by analysis of the integrated adult and juvenile thymus cells. **(D)** UMAP visualization of detection of TCR γ and/or δ (red), α and/or β (blue), or a combination of both sets of receptors (purple) in the integrated adult and juvenile thymi. **(E–G)** UMAP visualizations of the expression of ribosomal genes (E) *rps29*, (F) *rps15a*, and (G) *rplp1* that demonstrated differential expression in erythrocytes derived from juvenile vs. adult thymi as determined by Wilcoxon rank sum test in Seurat (Table S4).

To characterize the transcriptional landscape of the adult zebrafish thymus, we performed droplet-based scRNA-seq (10x Genomics) on live cells sorted from whole thymi derived from four zebrafish at 3–4 mo post-fertilization (mpf). SoupX was used to mitigate the impact of ambient mRNA contamination before quality-control filtering and sample integration in Seurat (Stuart et al., 2019; Young and Behjati, 2020). A total of 14,394 cells meeting the inclusion criteria were normalized by scTransform, integrated using reciprocal principal component analysis (PCA), clustered with the nearest neighbor approach, and visualized by uniform manifold approximation and projection (UMAP; Fig. 1 A and Table S1). Multiple stages of T cell development were evident: (1) rag1/2^+^ T cells, both cycling and non-cycling; (2) rag1/2^−^ cycling T cells; (3) *ipcef1*^+^ maturing T cells, with a distinct ccr7^+^ subpopulation; and (4) *il2rb*^+^ mature T cells (Fig. 1 B). Distinct subsets of T or T-like populations were also present: (1) T helper 2 (Th2) cells and/or type 2 innate lymphoid cells (ILC2s) expressing *gata3*, *il4*, and *il13*; (2) *gzmk*^+^, *gzma*^+^, *eomesa/b*^+^ cytotoxic T cells; and (3) γδ T cells expressing *sox13*, *trdc*, and *tcrg* (*si:dkeyp-13d12.23*). Additionally, many non-T cell populations were identified, including thymic epithelial cells (TECs; *epcam*^+^, *cdh1*^+^), dendritic-like (DC-like) cells (DC1, *spock3*^+^, *spi1a*^+^; DC2, *spock3*^+^, *ctsbb*^+^), macrophages *(havcr2*^+^, *mfap4*^+^), a mixed population composed largely of granulocytes (*mpx*^+^, *cpa5*^+^), natural killer–like (NK-like) cells (*eomesa*^+^, *fcer1gl*^+^), erythrocytes (*hbaa1*^+^, *hbaa2*^+^), and B cells (*pax5*^+^, *cd79a*^+^; Fig. S1 A). B cells were found to be predominately of the IgT isotype (previously named IgZ; Dornburg et al., 2021): 63% *ighz* cluster vs. 37% *ighd* cluster. Finally, we identified an ETP population expressing genes consistent with progenitor identity, including *si:dkey-261h17.1*, a *CD34* ortholog, and *csf1rb*, previously reported to be expressed in monocytes, macrophages, and some granulocytes in addition to hematopoietic stem and progenitor cells (HSPCs; Hason et al., 2022). Overall, these results reveal great cell type diversity within the zebrafish thymus, on par with mammalian thymic composition.

### Juvenile and adult thymi are largely composed of the same cell types but exhibit developmental differences in cellular composition and states

To assess how thymic composition changes over time, we analyzed juvenile thymi at 4 wk post-fertilization (wpf). Live cells were sorted from a pool of dissected thymi from 21 zebrafish and four technical replicates were sequenced (Fig. 1 C, Fig. S1 B, and Table S2). By increasing cell number by integrating cells from juvenile and adult thymi, we improved resolution and identified the following: (1) thrombocytes (*itga2b*^+^, *thbs1b*^+^); (2) fibroblast-like cells (*pdgfra/b*^+^, *col2a1a*^+^); and (3) *sox13*^−^ γδ T cells (*sox4a.1*^+^, *gata3*^+^, *etv5a*^−^; Fig. 1 D). Greater clarity was gained on the mixed granulocyte-rich population from the adult thymus composed of neutrophil-like granulocytes (*mpx*^+^, *npsn*^+^) and eosinophils (*gata2a*^+^, *viml*^+^; Fig. 1 D and Fig. S1 C). Seeing great overlap in the cell types present in the adult and juvenile thymi, we next asked whether cell type abundances were similar at both time points. We found the juvenile thymus was enriched in rag1/2^+^ T cells, rag1/2^+^ cycling T cells, and rag1/2^−^ cycling T cells (beta-binomial test; Fig. 1 E and Table S3). The juvenile thymus displayed greater joint expression of TCR α/β and γ/δ (Fig. S1 D). We also noted juvenile enrichments of sox13^+^ γδ T cells, sox13^−^ γδ T cells, and fibroblasts. Conversely, the adult thymus was enriched in maturing ccr7^−^ and ccr7^+^ T cells, TECs, and other non-T cell populations, including B cells, DC-like cells (1), mixed granulocytes, and macrophages. Examining global and cell-type specific gene expression between timepoints, we observed a striking difference in normalized expression of ribosomal subunit genes within adult and juvenile thymus erythrocytes, including that of *rps29*, *rps15a*, and *rplp1,* detected in <3–10% of juvenile erythrocytes vs. >85–91% of adult erythrocytes, with an average expression 12 to 20-fold higher in adult thymus erythrocytes (Fig. S1, E–G; and Table S4). These data show that although cell populations are similar between the adult and juvenile thymi, the cellular composition and transcriptional landscape of the zebrafish thymus does change over time, with adult thymi enriched in later-stage T cells and non-T cell populations and relatively fewer rag1/2^+^ T cells and γδ T cells.

### Zebrafish TECs share mammalian gene expression programs

**Figure S2.**
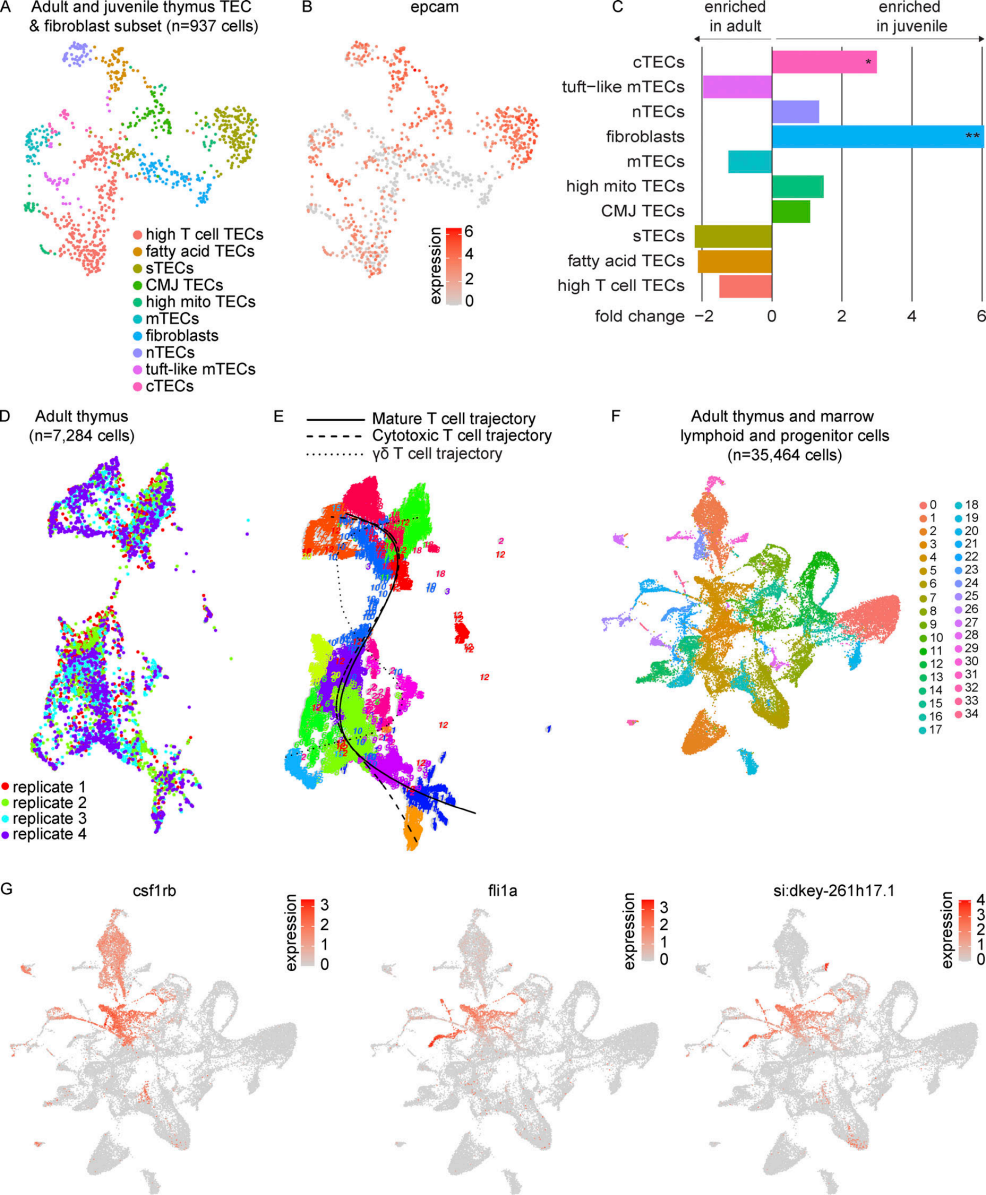
**Zebrafish TECs and T cell developmental trajectories. (A)** UMAP visualization of the 937 cells within the TEC and fibroblast populations of the combined adult and juvenile thymi as subsetted and re-analyzed to improve resolution of cell populations. Cell type annotations were based on differential expression analysis as determined by Wilcoxon rank sum test in Seurat (Table S5). **(B)** UMAP visualization of the expression of the epithelial marker, *epcam*, within the TEC and fibroblast subset. **(C)** Bar plot of beta-binomial test comparing composition of epithelial and fibroblast subset between juvenile and adult thymi (Table S6). Average fold change for each subpopulation is depicted by bar length and direction, with negative values enriched in the adult thymus and positive values enriched in the juvenile thymus. Statistical significance is shown for Benjamini-Hochberg adjusted P values: *, P < 0.05; **, P < 0.01. **(D)** UMAP visualization of RaceID3 clustering of cells derived from four adult thymi (*n* = 7,284 cells, independent analysis from Seurat) by replicate. **(E)** FateID principal curves fitted to mature T cell, cytotoxic T cell, and γδ T cell trajectories. **(F)** UMAP visualization of adult thymus and lymphoid and progenitor marrow fraction integrated together in Seurat (*n* = 35,464 cells) colored by cluster. These cells were derived from seven adult zebrafish dissected and processed in three independent experiments; paired marrow and thymi were obtained from two zebrafish as in Fig. 5, A–C. Cluster 4 is where the majority of thymus ETPs were highlighted in Fig. 5 C. **(G)** UMAP visualizations of marker genes used to identify the HSPC cluster (cluster 4). nTEC, neural TEC; mito, mitochondrial-gene expressing; CMJ, corticomedullary junction; sTEC, structural TEC.

To characterize the epithelial and stromal cells in greater detail, we re-integrated, clustered, and visualized the subset of *epcam*^+^ TECs and fibroblast-like cells (Fig. 1 F and Fig. S2, A and B; and Table S5). Analysis revealed 10 clusters, which we named in accordance with the mammalian literature (Baran-Gale et al., 2020). These populations included the following: (1) cortical TECs (cTECs; *psmb11a/b*^+^, *ccl25a*^+^); (2) medullary TECs (mTECs; *aire*^+^, *pvalb5*^+^); (3) structural TECs (*pros1*^+^, *col4a3*^+^); (4) neural TECs (*sstr5*^+^, *mal2*^+^); and (5) tuft-like mTECs (*prr15la*^+^, *fybb*^+^). We also noted fatty acid TECs (*elovl7a*^+^, *elovl6l*^+^) and a cluster consistent with corticomedullary junction TECs (*notch3*^+^, *krt5*^high^), expressing both medullary (*ccl19b*) and cortical chemokines (*ccl25a*, *cxcl12a*; Kadouri et al., 2020). Fibroblasts were distinguished from TECs by the lack of *epcam* expression and enrichment in *pdgfra/b*, *twist1a*, and *prrx1a/b*. By beta-binomial test, we observed an enrichment of fibroblasts in the juvenile thymus, consistent with our unsubsetted results, in addition to a smaller but significant enrichment of cTECs in the juvenile thymus (Fig. S2 C and Table S6). Our analysis highlights comparable diversity between zebrafish and mammalian TECs, with many putatively orthologous epithelial and stromal cell subtypes recognizable by conserved marker genes.

### Zebrafish T-cell development involves the equivalent of a CD4-CD8 double-positive state

**Figure 2.**
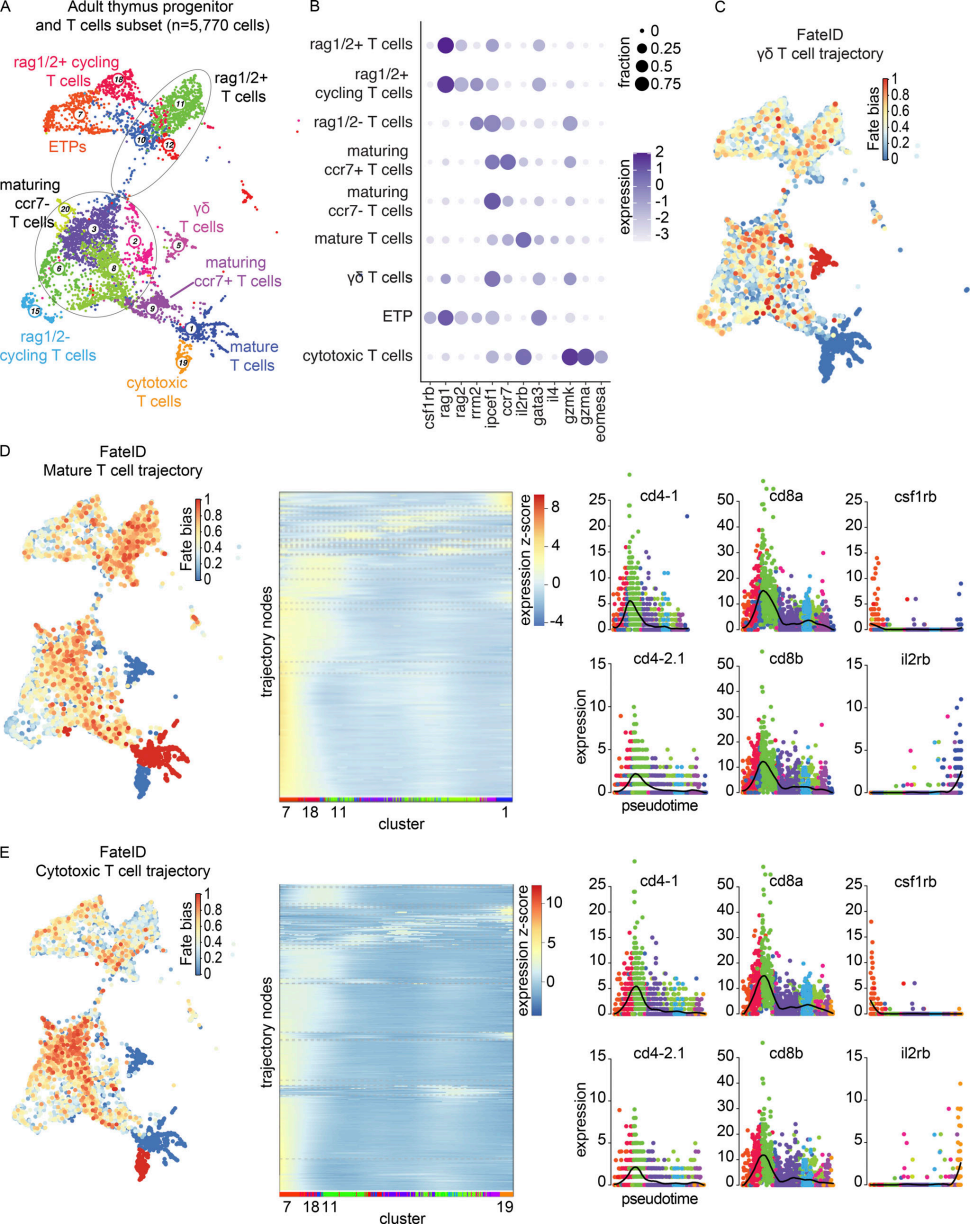
**Trajectory analysis of the adult thymus using FateID. (A)** RaceID3 UMAP visualization of the adult thymus progenitor and T cell subset. This subset of cells was derived from four adult zebrafish (3–4 mpf) dissected and processed within the same experiment. Cell type annotations were based on enriched markers and nomenclature consistent with Seurat clustering was used when appropriate. Circle outlines group multiple RaceID3 clusters falling within one Seurat-assigned cell type. **(B)** Dot plot of selected markers of the adult thymus progenitor and T cell subset showing expression across T cell populations. Dot size reflects the percentage of cells within a population expressing a given marker and dot color shows the log_2_ transformation of expression within that population. **(C)** Fate bias visualization depicts in color (red = high, blue = low) the probability that a cell will be assigned to the γδ T cell trajectory. Fate bias is 1 for the γδ T cell lineage-defining cluster, cluster 5, whereas clusters 1 and 19, lineage-defining clusters for the mature T cell and cytotoxic T cell trajectories, respectively, have a fate bias of 0. **(D)** Left: Fate bias visualization depicts in color (red = high, blue = low) the probability that a cell will be assigned to the mature T cell trajectory. Fate bias is 1 for the mature T cell lineage-defining cluster, cluster 1, whereas clusters 5 and 19, lineage-defining clusters for the γδ T cell and cytotoxic T cell trajectories, respectively, have a fate bias of 0. Middle: SOM for the mature T cell trajectory demonstrating pseudotime expression, grouping genes into nodes with RaceID3 cluster labeling along the x axis. Expression z-score is reflected by the color bar, with red being the highest and blue being the lowest. See also Table S7. Right: Pseudotime plots of the expression of *cd4-1*, *cd4-2.1*, *cd8a*, *cd8b*, *csf1rb*, and *il2rb* in the mature T cell trajectory. **(E)** Left: Fate bias visualization depicting in color (red = high, blue = low) the probability that a cell will be assigned to the cytotoxic T cell trajectory. Fate bias is 1 for the cytotoxic T cell lineage-defining cluster, cluster 19, whereas clusters 1 and 5, lineage-defining clusters for the mature T cell and γδ T cell trajectories, respectively, have a fate bias of 0. Middle: SOM for the cytotoxic T cell trajectory demonstrating pseudotime expression, grouping genes into nodes with RaceID3 cluster labeling along the x axis. Expression z-score is reflected by the color bar, with red being the highest and blue being the lowest. See also Table S7. Right: Pseudotime plots of the expression of *csf1rb*, *cd4-1*, *cd4-2.1*, *cd8a*, *cd8b*, *csf1rb*, and *il2rb* in the cytotoxic T cell trajectory.

**Figure 3.**
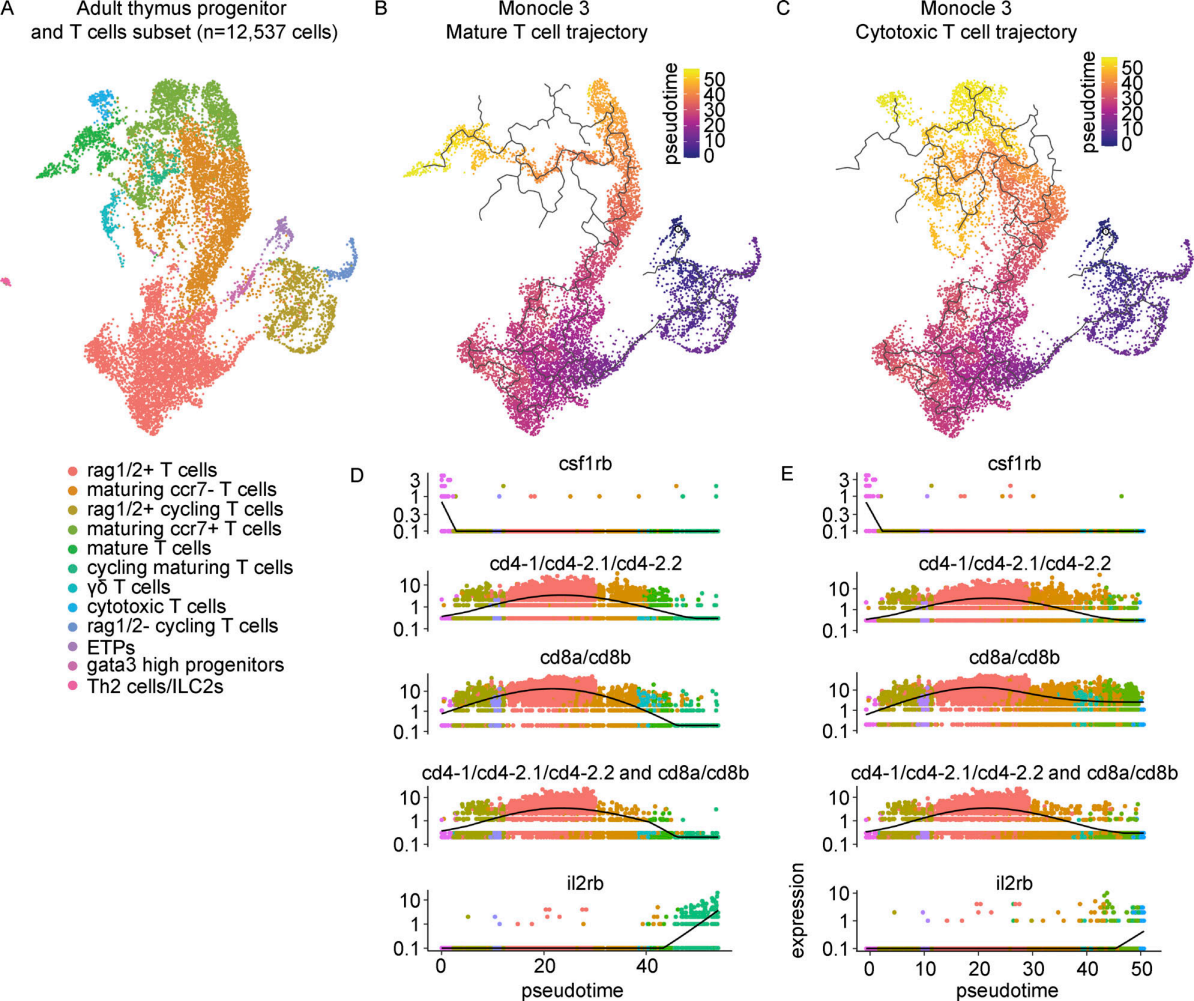
**Trajectory analysis of the adult thymus using Monocle 3. (A)** Monocle 3 UMAP visualization of the adult thymus progenitor and T cell subset. This subset of cells was derived from four adult zebrafish (3–4 mpf) dissected and processed within the same experiment. **(B and C)** UMAP visualization of the (B) mature T cell trajectory and (C) cytotoxic T cell trajectory colored by pseudotime as computed in Monocle 3. **(D and E)** Pseudotime plots of (top) *CD4* orthologs (sum of *cd4-1*, *cd4-2.1*, *cd4-2.2* expression), (middle) *CD8* orthologs (sum of *cd8a* and *cd8b* expression), and (bottom) dual *CD4* and *CD8* ortholog expression (minimum value of top and middle plots) in the (D) mature T cell trajectory and (E) cytotoxic T cell trajectory.

To investigate T-cell developmental trajectories, we performed FateID analysis (Herman et al., 2018) on a RaceID3 clustering of the adult thymus T cell populations in addition to Monocle 3 trajectory analysis (Cao et al., 2019). FateID analysis resolved three trajectories: trajectory 1 of the mature T cells, enriched in *il2rb* and *gata3* expression; trajectory 19 of the cytotoxic T cells, enriched in *gzma* and *eomesa* expression; and trajectory 5 of the γδ T cells (Fig. 2, A–E; and Fig. S2, D and E). We focused our downstream analysis on the more robustly inferred mature T cell (t1) and cytotoxic T cell (t19) trajectories. Self-organizing maps (SOMs) for t1 and t19 along with pseudotime plots of notable genes in T cell development revealed that the peak detection of *CD4* and *CD8* orthologs occurred within the rag1/2^+^ T cells with reduced levels of expression in both mature and cytotoxic T cells (Fig. 2, D and E, middle and right panels; and Table S7). Both trajectories demonstrated relatively high levels of *csf1rb* expression in the cluster assigned earliest in pseudotime (cluster 7, ETPs) and elevated levels of *il2rb* in the most mature populations (Fig. 2, D and E, right panels). These results were consistent with an independent trajectory analysis of the mature and cytotoxic T cells in Monocle 3 (Fig. 3, A–E). Furthermore, we identified cells that expressed at least one ortholog of *CD4* concomitantly with at least one ortholog of *CD8*, reminiscent of the CD4-CD8 double-positive state in mammals (Fig. 3, D and E). These “double-positive” cells were identified predominantly within the rag1/2^+^ T cells, rag1/2^+^ cycling T cells, and maturing ccr7^−^ T cells. To our knowledge, these results provide the first demonstration of *CD4*-*CD8* ortholog co-expression in zebrafish thymus at the single-cell level.

### ETPs retain myeloid gene expression but are enriched in T cell commitment genes

**Figure 4.**
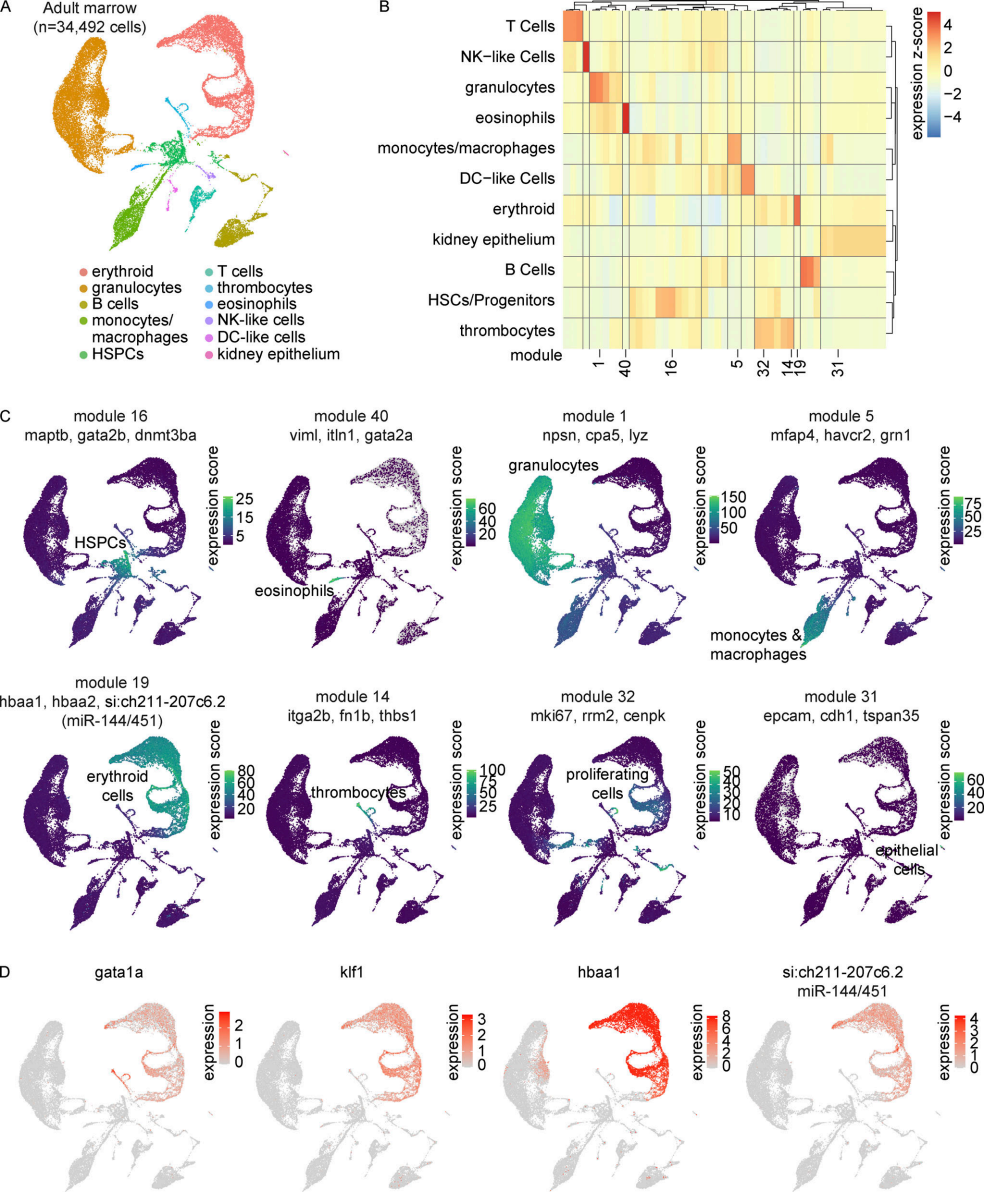
**Transcriptional characterization of the adult zebrafish marrow. (A)** UMAP visualization of 34,492 cells obtained from five zebrafish marrows. Marrows were dissected and processed in three independent experiments. Distinct cell lineages are denoted in different colors. Lineages were determined by differential gene expression (Wilcoxon rank sum test in Seurat) and gene module analysis in Monocle 3 (Table S8). **(B)** Gene module clustered heatmap of zebrafish marrow grouped by cell lineage. Color scale shows relative module enrichment (red) or depletion (blue). **(C)** UMAP visualizations of highlighted gene modules in panel B. **(D)** UMAP visualizations of canonical erythrocyte lineage genes *gata1a*, *klf1*, and *hbaa1* in addition to the newly appreciated *si:ch211-207c6.2*. This gene is one of the most specific erythrocyte genes identified at the transcriptional level in this dataset and was later determined to be a transcript containing the sequences of both *miR-144* and *miR-451*.

**Figure 5.**
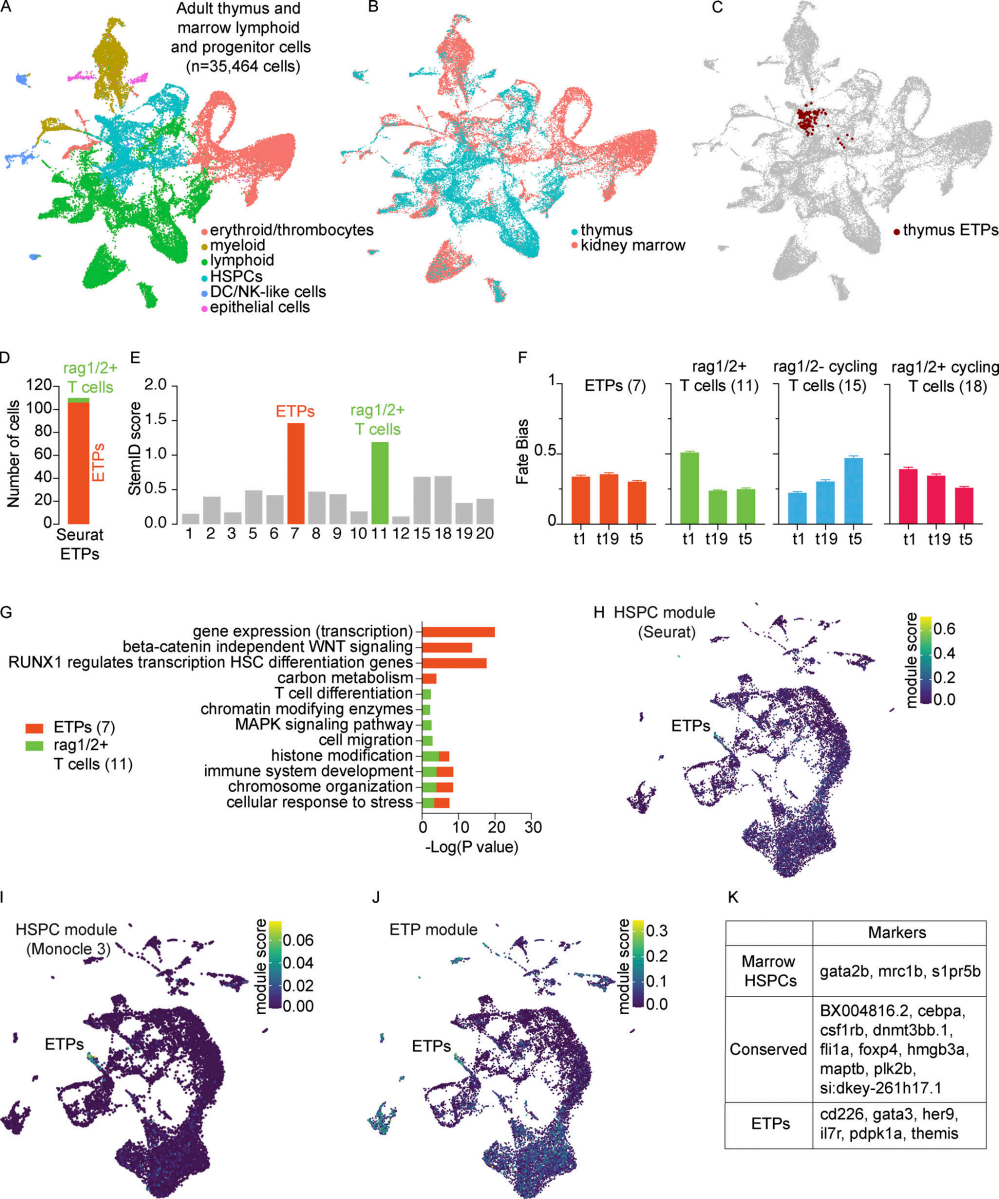
**Identification and characterization of ETPs. (A and B)** UMAP visualizations of the adult thymus and lymphoid and progenitor marrow fraction integrated together in Seurat (*n* = 35,464 cells) annotated by (A) cell lineage and (B) tissue of origin. These cells were derived from seven adult zebrafish dissected and processed in three independent experiments; paired marrow and thymi were obtained from two zebrafish. **(C)** UMAP visualization of Seurat ETPs (cluster 38, resolution = 3; Table S1) on the integrated adult thymus and lymphoid and progenitor marrow fraction UMAP. Highlighted cells fall predominately in the integrated HSPC cluster. **(D)** Assessment of the Seurat ETPs (cluster 38, resolution = 3) in RaceID3 analysis; the majority of Seurat ETPs were in RaceID3 cluster 7. **(E)** StemID score, the product of transcription entropy and number of inter-cluster links, displayed across all clusters; clusters 7 (ETPs) and 11 (rag1/2^+^ T cells) are highlighted in their respective cluster colors for having the highest StemID scores. **(F)** Fate bias probabilities within early clusters (highest StemID scores) across the three trajectories. Error bars depict SEM. **(G)** Enriched pathways determined using Metascape for the two earliest clusters (7 and 11) as predicted by FateID and StemID2. Negative log base 10 P values are plotted for a relevant subset of enriched pathways. **(H–J)** UMAP visualizations of gene modules in the adult thymus: (H) Seurat HSPC module (top 20 HSPC-enriched genes by specificity from Wilcoxon rank sum test), (I) Monocle 3 HSPC module, and (J) literature-based ETP gene module. See Materials and methods and Table S9 for module determination and the specific genes comprising each module, respectively. **(K)** Conserved and differentially expressed gene markers between thymic ETPs and marrow HSPCs as determined by Wilcoxon rank sum test (Tables S10 and S11). For the identification of conserved markers, P values were combined using Tippett’s method (minimum P value).

To better understand the earliest stages of T-cell development from HSPCs in the kidney marrow, we performed a large-scale scRNA-seq characterization of sorted populations from marrows of five adult (3–4 mpf) zebrafish. Consistent with previous work, from the 34,492 cells profiled, we identified cells from all major hematopoietic lineages, including erythrocytes, thrombocytes, granulocytes (neutrophil-like and eosinophil-like), monocytes, macrophages, T cells, B cells, and NK-like cells (Fig. 4, A–C; and Table S8). In addition to the canonical markers and cell populations previously reported, we identified *si:ch211-207c6.2* as an erythrocyte-specific pre-microRNA (pre-miRNA), *miRNA144/451* (Dore et al., 2008; Fig. 4 D) and recovered two populations of DC-like cells. Focusing specifically on characterizing the thymic ETPs and their relationship to marrow HSPCs, we next integrated cells from adult thymi and progenitor and lymphoid cells from adult marrows (*n* = 35,464 cells; Fig. 5, A and B). Thymic ETPs clustered in cluster 4, which was identified as HSPCs for its expression of *csf1rb*, *fli1a*, and a *CD34* ortholog (Fig. 5 C and Fig. S2, F and G). We additionally note that these cells characterized as ETPs in our Seurat analysis share great consistency with our RaceID3 ETP assignment (cluster 7; Fig. 5 D). StemID2 analysis revealed that cluster 7 had one of the highest StemID scores, greatest in clusters with high transcriptome entropy (i.e., uncertainty in differentiation) and connectedness to other clusters, consistent with the progenitor identity (Grun et al., 2016; Herman et al., 2018; Fig. 5 E). Cluster 7 was unbiased toward the three trajectories and was enriched in pathways related to hematopoietic stem cell differentiation but not T-cell differentiation like cluster 11 (rag1/2^+^ T cells), the other high StemID scoring cluster (Fig. 5, F and G). Further support for the successful identification of zebrafish ETPs came from the analysis of gene modules. Specifically, two independently determined HSPC modules from our marrow investigation (see Materials and methods) and a literature-based ETP gene list from mice and humans all had the greatest module scores in the cells we called ETPs (Fig. 5, H–J; and Table S9; Cante-Barrett et al., 2017; Cordes et al., 2022*Preprint*; Porritt et al., 2004; Rothenberg et al., 2008; Zeng et al., 2019). This evidence strongly suggests the successful identification of zebrafish ETPs in our analysis.

**Figure S3.**
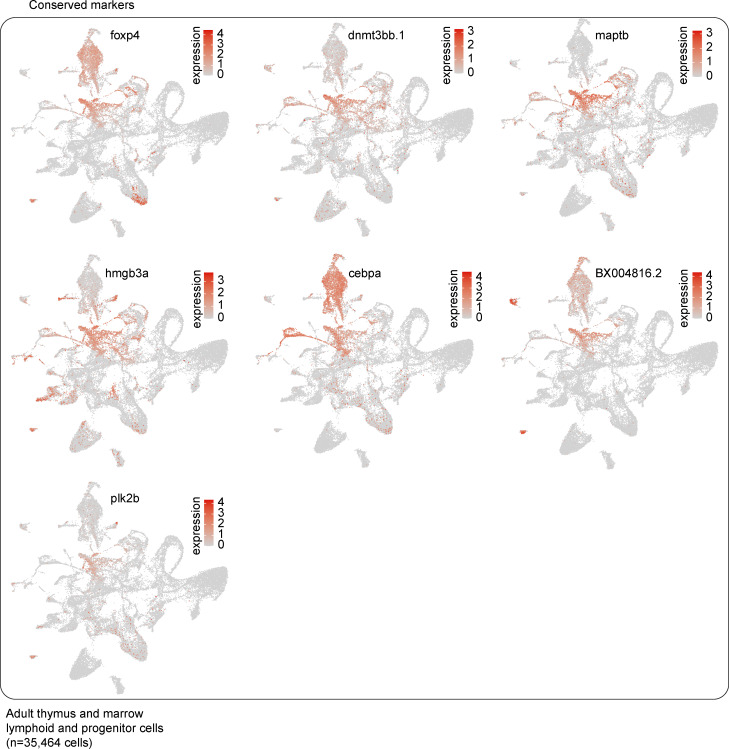
**Conserved genes between marrow HSPCs and ETPs.** UMAP visualizations of subset of genes identified as being conserved across thymic marrow HSPCs and ETPs; 7 of 10 conserved genes listed in Fig. 5 K are shown here as the other three conserved genes were visualized in Fig. S2 G (see also Table S10). Marker conservation was determined by Wilcoxon rank sum test; P values for the ETPs and HSPCs were combined using Tippett’s method (minimum P value).

**Figure S4.**
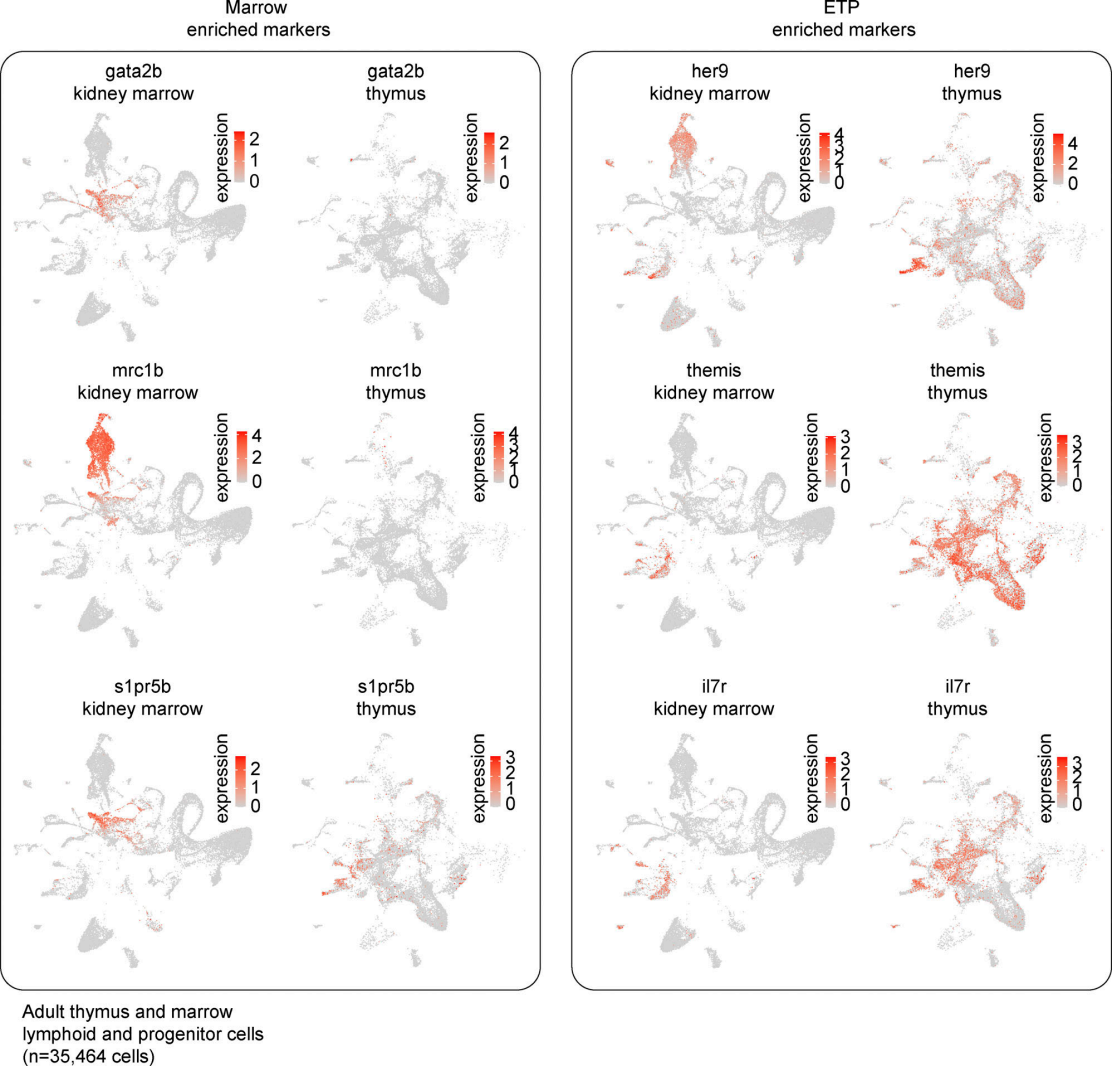
**Differentially expressed genes between marrow HSPCs and ETPs.** UMAP visualizations of subset of genes identified as being differentially expressed between marrow HSPCs and ETPs by Wilcoxon rank sum test (three shown for each; see also Table S11).

Having identified zebrafish ETPs, we next sought to compare the gene expression of these ETPs with marrow HSPCs. While analyzing and comparing the progenitor cells in these two hematopoietic organs is insufficient to inform us of the transitory states between marrow HSPCs and thymic ETPs, this comparison does allow us to identify changes in gene expression that occur at some point between these two states. This analysis identified 10 genes as being highly conserved (Fig. 5 K, Fig. S3, and Table S10). These genes included those whose mammalian orthologs have known stem cell functions like *plk2b*, which is highly expressed in aged hematopoietic stem cells and involved in regulating cell cycle G1 to S phase progression (Kowalczyk et al., 2015), *dnmt3bb.1* (Challen et al., 2014), *hmgb3a* (Nemeth et al., 2003), and the dual HSPC and myeloid lineage markers *cebpa* and *csf1rb* (Smith et al., 1996). Genes without a known connection to HSPC biology included *maptb*, whose human ortholog *MAPT* encodes microtubule-associated protein τ; *foxp4*; and the genomic region *BX004816.2*. Differential expression analysis revealed that kidney marrow HSPCs were enriched most prominently in *gata2b*, *s1pr5b*, and *mrc1b*, whereas thymic ETPs were enriched in T-cell commitment genes like *her9*, *themis*, *gata3*, *cd226*, *il7r*, and *pdpk1a* (Fig. 5 K, Fig. S4, and Table S11). Overall, these results transcriptionally define zebrafish ETPs and demonstrate that downregulation of *gata2b*, *s1pr5b*, and *mrc1b* with concomitant upregulation of early T cell commitment genes are features of this population.

### Large-scale kidney marrow analysis reveals distinct subpopulations, including multiple DC-like cell and NK-like cell subsets

**Figure 6.**
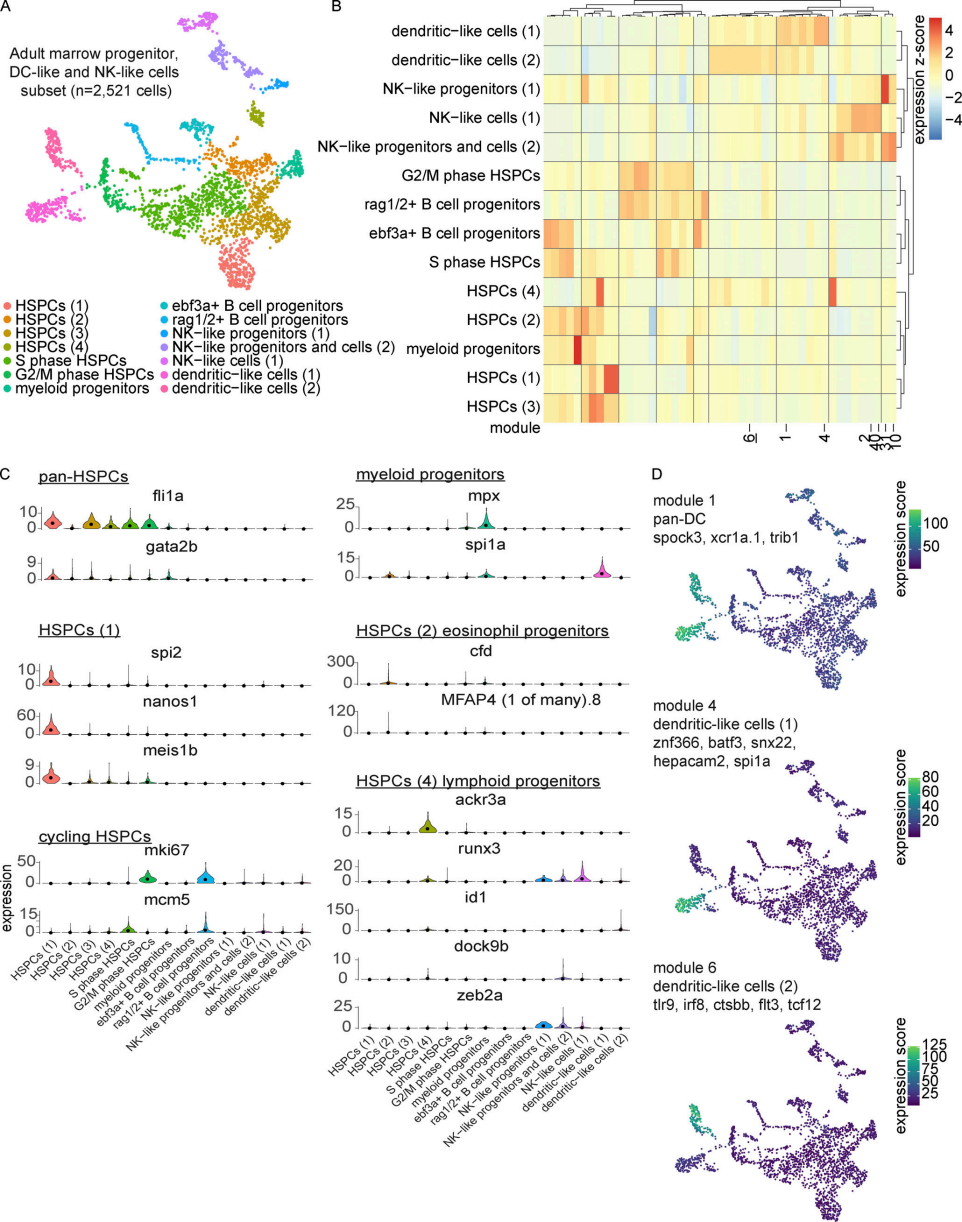
**Transcriptional characterization of marrow subset of HSPCs, DC-like cells, and NK-like cells. (A)** UMAP visualization of 2,521 DC-like cells, NK-like cells, and progenitors subsetted from the larger marrow UMAP. Cell type annotations were based on differential expression, as determined by Wilcoxon rank sum test in Seurat and gene module analysis in Monocle 3 (Table S12). **(B)** Gene module clustered heatmap of DC-like cells, NK-like cells, and progenitors grouped by cell type. Color scale shows relative module enrichment (red) or depletion (blue). **(C)** Violin plots of differentially expressed genes as determined by Wilcoxon rank sum test among HSPC clusters. **(D)** UMAP visualizations of gene modules enriched across all DC-like cell populations (module 1) and specific to either the cDC population (DC-like cells [1]; module 4) or the plasmacytoid-like DC population (DC-like cells [2]; module 6).

**Figure 7.**
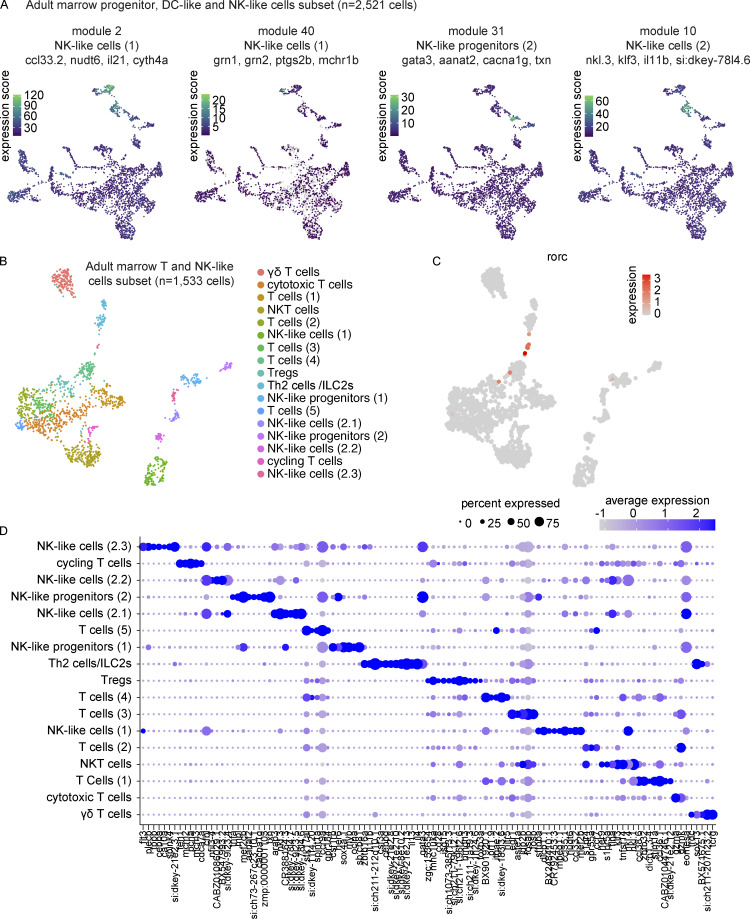
**Transcriptional characterization of marrow NK-like and T cell subpopulations. (A)** UMAP visualizations of gene modules enriched across NK-like populations in the progenitor, DC-like cell, and NK-like cell marrow subset. **(B)** UMAP visualization of 1,533 T and NK-like cells as subsetted, re-integrated, and clustered from the larger marrow UMAP in Fig. 4 A. Cell type annotations were based on differential expression analysis as determined by Wilcoxon rank sum test in Seurat (Table S13). **(C)** UMAP visualization highlighting *rorc*^+^ T cells, too small a population to be resolved as an independent cluster, but nevertheless demonstrating clear heterogeneity. **(D)** Dot plot of selected markers differentially expressed across T and NK-like cell populations. Dot size reflects the percentage of cells within a population expressing a given marker and dot color shows the average expression within that population.

Having characterized zebrafish T-cell development in the thymus, we were next interested in returning to our large-scale characterization of the zebrafish marrow. Intrigued by the identification of two populations of DC-like cells and what appeared to be a heterogenous NK-like cell population, we performed gene module analysis in Monocle 3 on the marrow subset of progenitors, DC-like cells, and NK-like cells (Fig. 6, A–C and Table S12). This analysis revealed great heterogeneity within HSPC populations (*fli1a*^+^, *gata2b*^+^) and identified multiple subsets, including, HSPCs (1; *nanos1*^+^, *spi2*^+^), HSPCs (2)/eosinophil progenitors (*cfd*^+^, *MFAP4 (1 of many).8*^+^), cycling HSPCs (*mki67*^+^ or *mcm5*^+^), and HSPCs (4)/lymphoid progenitors (*ackr3a*^+^, *runx3*^+^; Fig. 6 C). The myeloid progenitor and HSPCs (4)/lymphoid progenitor expression patterns were particularly interesting for their shared expression of genes with the DC-like cells (1) and DC-like cells (2) populations, respectively. We identified both pan-DC cell modules and subset specific modules suggestive of significant gene expression differences between these populations (Fig. 6 D). Pan-DC markers included *spock3*, *xcr1a.1*, and *trib1*, whereas subset analysis suggested the existence of both conventional-like DCs (cDCs), specifically cDC1s expressing *znf366*, *batf3*, *snx22*, and *hepacam2,* and plasmacytoid-like DCs enriched for endosomal TLRs (e.g., *tlr9*), *irf8*, *ctsbb*, *flt3*, and *tcf12* (Fig. 6 D). Gene module analysis for the NK-like cells revealed distinct sets of genes in multiple NK-like cell states, e.g., NK-like cells (1), enriched in *ccl33.2*, *nudt6*, *il21*, and *cyth4a* (Fig. 7 A). A very small subcluster of this population appeared to be *eomesa*-negative and was enriched in the macrophage/DC-like markers *grn1*, *grn2*, *ptgs2b*, and *mchr1b* (module 40). Additionally, although not separate clusters at this resolution, distinct gene sets were found within the NK-like progenitors and cells (2), separating into progenitors (2; module 31) and NK-like cells (2; module 10). These findings provide valuable insight into the great transcriptional heterogeneity within understudied zebrafish immune cell populations and prime future investigations.

### Zebrafish T and NK-like cells are distinguished transcriptionally by multiple genes including the *SPI1*/*PU.1* ortholog, *spi1b*

**Figure 8.**
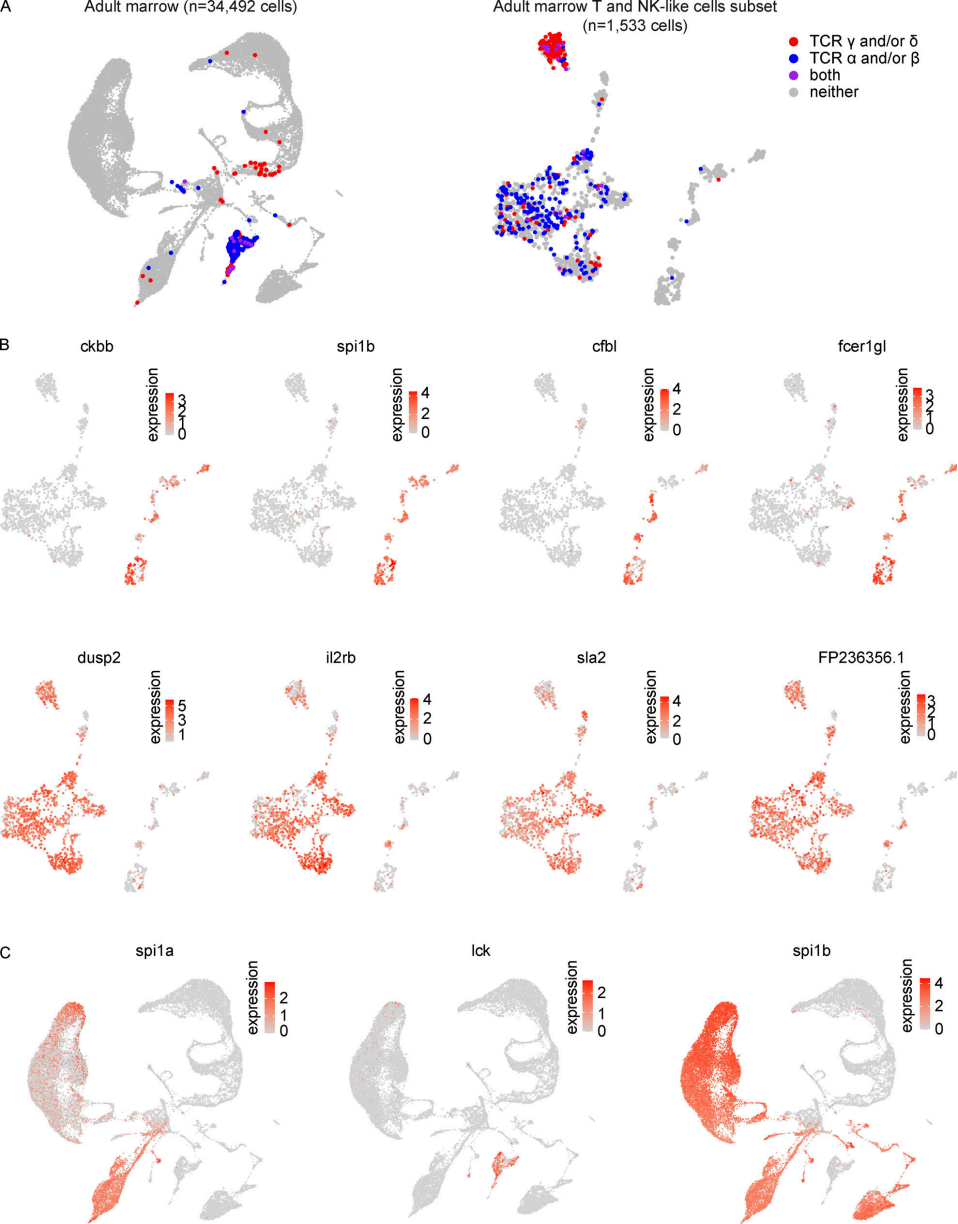
**Distinguishing markers of NK-like cells and T cells. (A)** UMAP visualization of detection of TCR γ and/or δ (red), α and/or β (blue), or a combination of both sets of receptors (purple) in the (left) larger marrow object and (right) T and NK-like cell populations. **(B)** Top: UMAP visualizations of subset of genes identified in both Monocle 3 top markers analysis (limited to top 25 most specific genes as ranked by Jensen-Shannon divergence) and Seurat differential expression analysis (Wilcoxon rank sum test) as being significantly enriched in NK-like cells vs. T cells (Table S14). Bottom: UMAP visualizations of subset of genes identified in both Monocle 3 top markers analysis and Seurat differential expression analysis as being significantly enriched in T cells vs. NK-like cells. *FP236356.1* is a 109,805 bp region that includes TCR β constant 1 (*trbc1*; Table S14). **(C)** UMAP visualizations of expression of *spi1a*, *spi1b*, and *lck* in the marrow. Expression of *spi1a* is observed in a subset of *spi1b-*expressing cells and may distinguish myeloid and lymphoid cells. NK-like cells here are *spi1b*^+^ while T cells are *spi1b* negative. DC-like cells (2) are *spi1a*^−^*lck*^+^ whereas DC-like cells (1) are *spi1a*^+^*lck*^−^, further consistent with the hypothesis that DC-like cells (2) are plasmacytoid DCs and DC-like cells (1) are myeloid DCs and specifically akin to cDC1s.

To explore NK-like cell biology in greater detail and to specifically relate these innate lymphoid cells to their T-cell counterparts, we subsetted all marrow NK-like cells and T cells (Fig. 7 B and Table S13). This analysis revealed rich diversity of T and NK-like states and demonstrated great consistency with our previous NK-like cell analysis, thereby allowing similar nomenclature to be used (Fig. 7, C and D). In addition to the diversity appreciated at the cluster level, we also noted a small population of *rorc*+ lymphocytes that appeared to be a distinct population (Fig. 7 C). To better support the assignments of these populations as T- and NK-like cells, we first assessed the expression of TCR α/β/γ/δ chains in these cells in the full marrow and a close-up analysis of this subset (Fig. 8 A). In the subset, we noted a mix of αβ- and γδ-expressing T cells in the main T-cell cluster, distinguished from a smaller side cluster of cells predominantly expressing γ and/or δ chains. In total, within the clusters deemed to be T cells, 365 out of 1,244 cells (29.3%) expressed at least one TCR gene, on par with the detection efficiency of other T-cell associated genes like *lck* (detection efficiency = 27.4%). This contrasted with the rare recovery of these TCR transcripts in the clusters denoted as NK-like cells, with only 3 out of 289 cells (1%) expressing any of these transcripts. Further investigation into the transcriptional differences between these populations revealed specific enrichments in each population, including *ckbb*, *spi1b*, *cfbl*, and *fcer1gl* in NK-like cells and *dusp2*, *il2rb*, *sla2*, and *FP236356.1* in T cells (Fig. 8 B and Table S14). To contextualize these results, we note that innate lymphoid cells (e.g., NK-like cells) here were *lck*^−^, *spi1b*^+^, and *spi1a*^−^, whereas T cells were *lck*^+^, *spi1b*^−^, and *spi1a*^−^ (Fig. 8 C). These data identify key distinguishing markers between marrow NK-like cells and T cells in zebrafish.

### B lymphocyte development demonstrates transcriptional conservation in key developmental genes and includes a pre-B cell state

**Figure 9.**
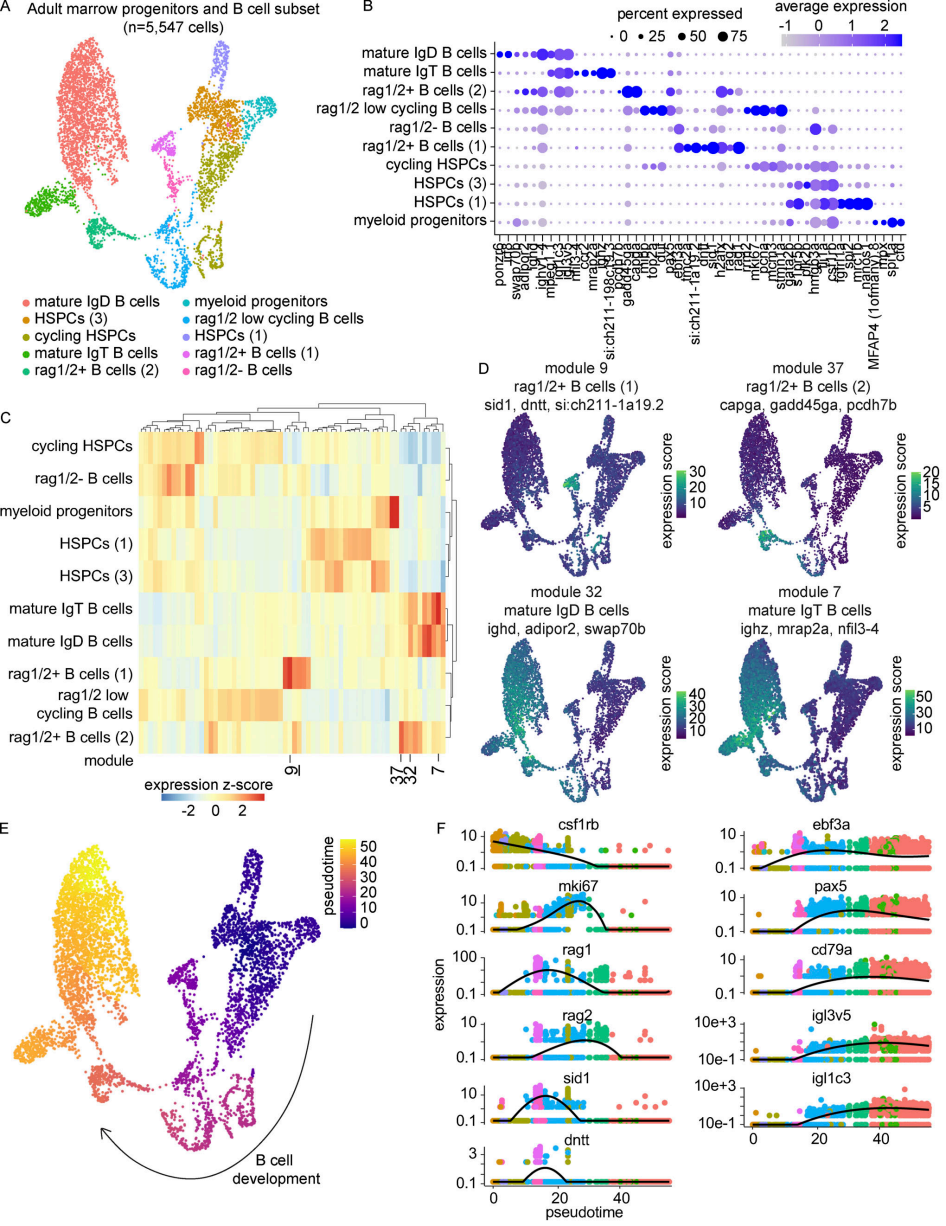
**Transcriptional characterization and trajectory analysis of B cell development using Monocle 3. (A)** UMAP visualization of 5,547 B cells and progenitors from the marrow. Cell type annotations were based on differential expression as determined by the Wilcoxon rank sum test in Seurat and gene module analysis in Monocle 3 (Tables S15 and S16). Nomenclature consistent with Fig. 6 A was used for the HSPC populations when appropriate. **(B)** Dot plot of selected markers differentially expressed across B and progenitor cell populations. Dot size reflects the percentage of cells within a population expressing a given marker and dot color shows the average expression within that population. **(C)** Gene module clustered heatmap of zebrafish B cells and progenitors grouped by cell type demonstrating cell type enrichments. Color scale shows relative module enrichment (red) or depletion (blue). **(D)** UMAP visualizations of gene modules enriched in different stages of B cell development. **(E)** UMAP visualization of B cells and progenitors colored by pseudotime as computed in Monocle 3. **(F)** Gene expression dynamics over pseudotime computed in Monocle 3 colored by cluster assignment for 11 genes related to B cell development.

To investigate zebrafish B cell development, we subsetted B and progenitor cells from the marrow for further analysis (Fig. 9 A). HSPC populations, multiple stages of B cell development, and two mature B cell populations (IgT and IgD) were distinguished by differential gene expression (Fig. 9 B and Table S15). Relative to the thymus, marrow IgT B cells only comprised 15% of mature B cells (vs. 63%). Of note, while *ighd* and *ighz* were detected in all B cell populations, including rag1/2^+^ B cells (1) and rag1/2^−^ B cells, light chain genes *igl1c3* and *igl3v5* were not detected in these two populations (Fig. 9 B). Gene module analysis identified additional genes enriched at different stages of B cell development (Fig. 9 C and Table S16). Module 9, expressed in rag1/2^+^ B cells (1), included the genes *dntt*, *sid1*, and *si:ch211-1a19.2*, the former encoding the enzyme terminal deoxynucleotidyl transferase, important in generating junctional diversity in V(D)J recombination, and the latter two containing immunoglobulin domains (Alt and Baltimore, 1982; Komori et al., 1993; Yoder et al., 2002; Fig. 9 D). Later developing B cells were enriched in migratory genes including *capga*, *gadd45ga*, and *pcdh7b* (module 37). Additional genes found to be differentially expressed between mature IgD and IgT B cells included *adipor2* and *swap70b* enriched in module 32 and *mrap2a* and *nfil3-4* enriched in module 7 (Fig. 9 D). These data illuminate great transcriptional heterogeneity throughout zebrafish B cell development and show differences in thymic and marrow mature B cell isotype abundances.

**Figure 10.**
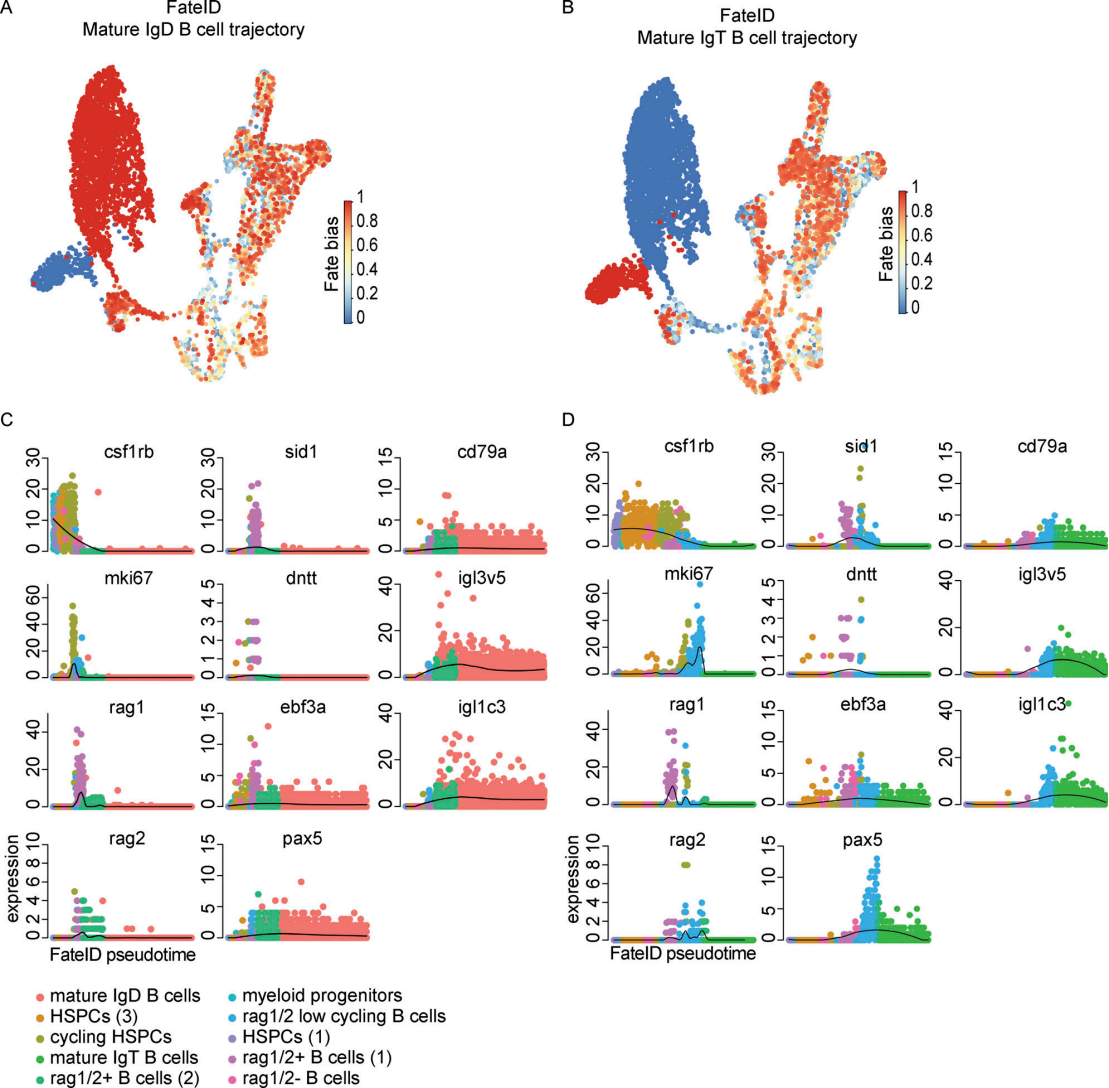
**Trajectory analysis of B cell development using FateID. (A and B)** UMAP visualizations where fate bias is depicted in color (red = high, blue = low) and represents the probability that a cell will be assigned to a given lineage. The lineage-defining clusters (A) mature IgD B cells and (B) mature IgT B cells have a fate bias of 1 in their respective trajectory maps and a fate bias of 0 in the other. **(C and D)** Pseudotime plots of the expression of *csf1rb*, *mki67*, *rag1*, *rag2*, *sid1*, *dntt*, *ebf3a*, *pax5*, *cd79a*, *igl3v5*, and *igl1c3* in the (C) mature IgD B cell trajectory and (D) mature IgT B cell trajectory.

Interested in the differential detection of heavy and light chain genes, we investigated the transcriptional dynamics of B cell development in greater detail through Monocle 3 pseudotime analysis (Fig. 9, E and F). We selected 11 genes that would allow us to explore lineage commitment (*csf1rb*, *ebf3a*, *pax5*, *cd79a*), B-cell receptor rearrangement (*rag1*, *rag2*, *sid1*, *dntt*), light chain expression (*igl3v5*, *igl1c3*), and cell state (*mki67*). Specifically focused on the timing of B cell heavy and light chain gene expression where IgD and IgT B cells would not be expected to differ greatly, we chose to explore gene expression in pseudotime for B cell development as a whole and not along any particular branched structure. We observed a concomitant decrease in *csf1rb* expression with increase in *ebf3a* expression at the transition between multipotent progenitor and commitment to the B cell lineage. Multiple stages of proliferation were evidenced by *mki67* expression, the first at the juncture of B cell commitment and the second following a phase of *sid1*, *dntt*, and *rag1/2* gene expression. The expression of the B cell genes *pax5* and *cd79a* followed that of the expression of *ebf3a* and they remained expressed onward in development. The expression of r*ag1/2* was noted in two distinct populations of B cells, with rag1/2^+^ B cells (1), the earlier population in pseudotime, enriched in *sid1* and *dntt* expression and notably negative for immunoglobulin light chain expression. While largely overlapping, the detection of *rag1* and *rag2* expression was not identical, with *rag2* expression remaining elevated in rag1/2 low cycling B cells. An analogous analysis using FateID revealed similar pseudotime assignment and gene expression dynamics throughout B cell development (Fig. 10, A–D). The most noticeable differences in these analyses were the relative ordering of HSPC populations, which were not relevant to our consideration of B cell development post-commitment. Taken together, these results show the dynamic gene expression changes that occur in zebrafish B cell development and strongly support the existence of a heavy chain^+^ and light chain^−^ pre-B cell state.

## Discussion

scRNA-seq has proven to be a powerful technique not only for revealing cell type and tissue heterogeneity but also inferring cellular developmental trajectories. By profiling ∼70,000 total cells from the zebrafish primary lymphoid organs, the kidney marrow and thymus, we painted a high-resolution picture of zebrafish lymphocyte development. Beginning with the zebrafish thymus, an understudied organ, we explored two different timepoints of T cell development and noted substantial cell type similarity but finer differences in thymic composition and gene expression. We selected the early timepoint of 4 wpf as the earliest feasible dissection of a morphologically mature thymus (Lam et al., 2002; Willett et al., 1997b), balancing desired cell number, dissection quality, and cellular preparation efficiency. While we anticipated many of the T-cell developmental changes that we observed, namely the relative decrease in rag1/2^+^ developing T cell subsets over time consistent with involution, we were surprised that the juvenile thymi contained a similarly rich array of non-T cell populations from DC-like cell subsets to NK-like cells. The presence, albeit low, of thymic B cells at 4 wpf was particularly interesting as mature B cells are only first detected ∼3 wpf (Page et al., 2013). The observation that erythrocytes detected in juvenile and adult thymic samples not only differed in embryonic vs. adult hemoglobin expression but also in ribosomal subunit expression suggests that there may be temporal ribosomal heterogeneity (Kondrashov et al., 2011). Zebrafish TECs exhibited rich diversity, transcriptionally reminiscent of multiple mammalian subsets including mTECs, cTECs, and tuft-like mTECs. Although not entirely consistent transcriptionally due to the lack of *psmb11a* and *psmb11b* detection, our high T cell TECs could represent the zebrafish equivalent of mammalian thymic nurse cells (Nakagawa et al., 2012). Thymic fibroblasts appeared to be activated, as suggested by the expression of genes commonly expressed in cancer-associated fibroblasts, *twist1a* and its downstream transcription factor *prrx1a/b* (Yeo et al., 2018). We note that these findings, like all others of this study, are interpreted in relation to the assumed roles of these genes in zebrafish based on known mammalian biology and that there may be species-specific differences that account for such described transcriptional “inconsistencies.” We speculate that these results only touch the surface of zebrafish TEC heterogeneity due to our lack of enzymatic digestion of the thymus tissue and believe this could be an exciting area of future research. ETPs were identified by their expression of early T cell differentiation genes and retention of some HSPC markers. Notably, *gata2b*, one of the best markers of marrow HSPCs, was detected in <2% of thymic ETPs, potentially reflecting an early and required downregulation to commit to the T cell lineage. The subset-specific markers identified here provide a springboard for future studies to visualize early T-cell development including T cell-TEC/T cell–fibroblast interactions through the generation of ETP-, TEC-, and fibroblast-specific transgenic lines and provide a larger window into the evolution of T-cell immunity and T-cell development.

Building upon prior transcriptional characterizations of the zebrafish marrow, our work highlights the existence of at least two DC-like subsets and extensive heterogeneity within the marrow T, NK-like, and B cell populations. The two DC-like populations seem to reflect a lymphoid-myeloid split, with the population identified as resembling plasmacytoid DCs expressing a lymphoid signature and the type 1 cDC-like population expressing a myeloid signature. Intriguingly, an *XCR1* ortholog was identified as a pan-zebrafish marrow DC marker, while it has been reported to be an exclusive cDC1 marker in mice (Bachem et al., 2012). Zebrafish T and NK-like cell diversity within the marrow richly paralleled that of mammals with recovery of transcriptional profiles consistent with cytotoxic T cells, NKT cells, Th2s/ILC2s, regulatory T cells, multiple subsets of NK-like cells, and even a small population of *rorc*^+^ TCR-lacking ILC3s. TCR expression clearly distinguished T and innate lymphoid cells such as NK-like cells in our data. Although *il2rb* has been reported to be a marker of NK cells in past studies, our results strongly suggest *il2rb* as a marker of mature thymic and marrow T cells (Carmona et al., 2017; Tang et al., 2017). Our B cell development investigations show that zebrafish *ebf3a* may act like mammalian *EBF1* (Hagman et al., 1991) as a B cell lineage commitment factor. Pseudotime analysis tracing the development of B cells from multipotent progenitors to mature B cells further revealed intriguing findings. The presence of a *sid1*^+^, *dntt*^+^, *rag1/2*^+^, *igl3v5*^−^, *igl1c3*^−^ population of developing B cells is highly suggestive of the existence of a pre-B cell state. Although underexplored, *sid1* has been proposed as an ortholog to *VPREB1* (Yoder et al., 2002), which would be consistent with this finding. We also speculate whether the gene *si:ch211-1a19.*2 may encode an additional surrogate light chain subunit and perhaps be a λ5 homolog, as it has been classified previously (Garcia et al., 2018). Collectively, our work provides a rich transcriptional atlas of the zebrafish marrow and thymus and sets the stage for future investigations to better understand teleost adaptive immunity and the immunological modeling of human disease in zebrafish.

## Materials and methods

### Zebrafish husbandry and lines

Zebrafish were maintained in accordance with Boston Children’s Hospital Institutional Animal Care and Use Committee protocols and in line with Animal Resources at Children’s Hospital guidelines. In total, seven adult zebrafish (3–5 mpf) and 21 juvenile zebrafish (4 wpf) were analyzed in these studies. Five of the adult zebrafish were generated through the mating of a pTol2-hsp70l:Cas9-t2A-GFP, 5xU6:sgRNA guide fish with a pTol2-DRv7 GESTALT (Genome Editing of Synthetic Target Arrays for Lineage Tracing) barcode fish (McKenna et al., 2016; Raj et al., 2018b). The parental strains were gifts from the Schier and Gagnon labs, respectively (Harvard University, Cambridge, MA; University of Utah, Salt Lake City, UT). Two-stage barcoding was induced in these fish, first by injection at the single-cell stage with sgRNAs 1–4 and again at 28 h after fertilization by heat shock as previously described (Raj et al., 2018a). The remaining two adult fish and all 21 juvenile zebrafish were Tg(*lck:eGFP*), utilized for the purpose of identifying and isolating T cells, as previously described (Langenau et al., 2004). Of the adult fish, six were female and one was male. Sex of the juvenile fish could not be determined at the time of analysis.

### Tissue collection and cell sorting

**Figure S5.**
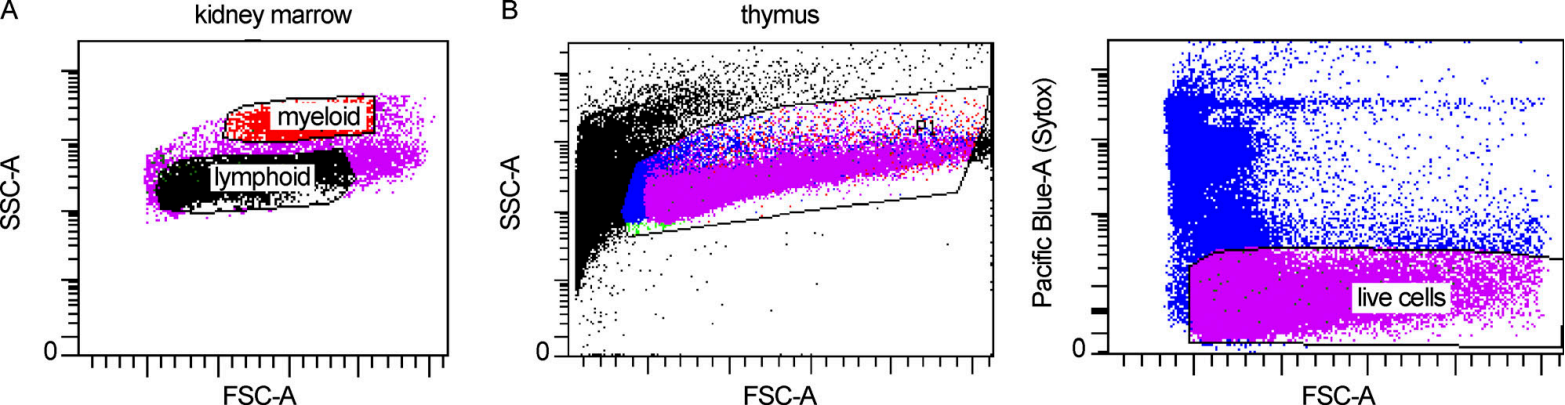
**Fluorescence-activated cell sorting gates for marrow and thymus. (A and B)** Representative fluorescence-activated cell sorting gating for (A) kidney marrow and (B) thymus sorts. The lymphoid gate of the marrow encompasses both lymphoid and progenitor cells whereas the myeloid gate contains mostly granulocytes. All live, single cells were sorted from the thymi. SSC-A, side scatter area; FSC-A, forward scatter area.

Adult and juvenile zebrafish were euthanized by rapid chilling and confirmed deceased prior to dissecting thymi and/or kidney marrows. To reduce erythrocyte contamination from the peripheral blood, adult fish were cardiac bled prior to tissue dissection using a heparin-coated (1,000 USP U/ml in 1× [PBS) p10 or p20 pipette tip. The juvenile fish were deemed too fragile for this procedure to be performed. For the thymi dissections, the zebrafish were positioned on their sides on a Styrofoam lid under a fluorescent dissecting scope and held in place/braced with the aid of one 23-gauge needle and gentle counterpressure applied with the aid of dissection scissors and/or forceps. The operculum was then removed, allowing access to and removal of one of the thymi, and placed in room temperature 0.5% BSA in HBSS without Ca^2+^ and Mg^2+^. Each fish was then carefully flipped over to the other side and this procedure was repeated to obtain the second thymus. When fluorescent reporters were not employed, brightfield microscopy and anatomical landmarks were used instead. Kidney marrows were dissected under a dissecting microscope by placing the zebrafish on a paper towel and using dissection scissors to make a superficial incision along the ventral midline to expose the internal organs. Forceps were used to remove all exposed organs including the intestines, liver, and swim bladder. In female zebrafish, eggs were also removed to clear the surgical field. Once visible along the dorsum, the kidneys were removed using forceps and placed in 0.5% BSA in DPBS without Ca^2+^ and Mg^2+^ on ice. In the case of joint thymus and kidney marrow dissection (two adult fish), the thymi were dissected first. The tissues were then mechanically dissociated into single-cell suspensions by pipetting and filtered through a 50-μm disposable filter (CellTrics). The filtered samples were then briefly centrifuged at 400 *g* × 5 min to remove debris and resuspended in their respective buffers for flow cytometry. Sytox blue was added to the samples immediately prior to analysis to assess cell viability. Live cells were sorted (85 μm nozzle) on a BD FACSAriaII into 300 μl of 0.5% BSA in DPBS and kept on ice following the sort. In the case of the marrows, live cell sorts were divided into two fractions based on forward scatter and side scatter characteristics—a lymphoid and progenitor fraction and a granulocyte fraction (Fig. S5, A and B). This allowed for the relative enrichment of lymphoid and progenitor cells in the overall analysis, advantageous for our goal to investigate B cell developmental trajectories. By nature of this sorting strategy, our marrow data does not reflect whole kidney marrow composition. Between 50,000 and 100,000 cells were sorted per sample.

### scRNA-seq and genome alignment

Sorted cells were pelleted by centrifugation at 400 *g* × 5 min and resuspended to a final concentration of 1,000 cells/μl following Trypan blue staining. Transcriptome profiling was performed using the Chromium Next GEM Single Cell 3′ v3.1 Reagent Kits (10x Genomics). A targeted recovery of 6,000 cells was set for loading the Chromium Controller in all the assays, and steps were performed as described in the Chromium Next GEM Single Cell 3′ Reagent Kits v3.1 User Guide (Rev D). The final transcriptome libraries were pooled in sets of 4 or 5 and sequenced at the Harvard University Bauer Core (https://bauercore.fas.harvard.edu/) on an Illumina NovaSeq 6000 using an SP flow cell (Read 1: 28 cycles, i7 index: 8 cycles, i5 index: 0 cycles, Read 2: 91 cycles). For transcriptome analysis, demultiplexed FASTQ files were processed through the Cell Ranger 3.1.0 pipeline (Zheng et al., 2017) and aligned to a Zon lab custom genome. More specifically, this Zon lab custom genome consisted of zebrafish Ensembl GRCz11 and five additional genes (EGFP, mCherry, dsRed, TagBFP, and BRAFv600e). This was done for consistency and to retain the ability to assess EGFP expression from the *lck:eGFP* zebrafish. (Note: BRAFv600e denotes the activating missense mutation in human B-Raf proto-oncogene [BRAF] commonly seen in some cancers. This gene is included as part of our standard alignment procedure when recovery of EGFP is desired. At processing, the BRAFv600e “gene” contained both the BRAFv600e sequence in addition to part of the SV40 poly(A) signal also found in the *lck:eGFP* plasmid. For this reason, “BRAFv600e” is recovered in this dataset in a similar set of cells where EGFP is identified.)

### Quality control and data processing in SoupX and Seurat

Unfiltered feature-barcode matrices from the Cell Ranger outputs for all samples were loaded into the SoupX package (Young and Behjati, 2020) to create SoupChannel objects. Multiple methods of contamination fraction estimation were applied and assessed qualitatively for success of contamination removal and sparing of true signal using built-in functions. SoupX processing required a more recent version of Seurat (Stuart et al., 2019) to be used. Therefore, Seurat library v3.2.2 was attached prior to the analysis. Seurat v3.1.3 was used for all other analyses. The following contamination fraction settings were used for each sample: all four adult thymus datasets ρ = 0.02; lymphoid 27, myeloid 27 ρ = 0.05; lymphoid 22, myeloid 22, lymphoid 24, myeloid 24, all four juvenile thymus datasets ρ = 0.07; myeloid 44, myeloid 49 ρ = 0.10; lymphoid 44, lymphoid 49 ρ = 0.18. The corrected count matrices were then loaded into Seurat v3.1.3. This included the application of quality control filters (see below), normalization (scTransform; Hafemeister and Satija, 2019), finding integration anchors (reciprocal PCA), and integrating the data. Metadata on tissue of origin (marrow vs. thymus) or age of fish (juvenile vs. adult) were stored when useful.

The following quality control filters were applied: (1) Adult thymus: At least 200 genes but no more than 4,000, at least 500 UMIs but no more than 25,000, and a mitochondrial gene percentage <15%; (2) Integrated adult and juvenile thymi: At least 200 genes but no more than 4,000, at least 500 UMIs but no more than 30,000, and a mitochondrial gene percentage <15%; (3) Lymphoid/progenitor and myeloid marrow fractions: At least 200 genes but no more than 4,000, at least 1,500 UMIs but no more than 50,000, and a mitochondrial gene percentage <15%. A more stringent lower bound on UMIs was applied here than on other objects due to clusters appearing to contain cell fragments at lower cutoffs that affected cell characterization; (4) Integrated lymphoid/progenitor marrow fraction and adult thymus: At least 200 genes but no more than 4,000, at least 500 UMIs but no more than 38,000, and a mitochondrial gene percentage <15%.

Following integration, standard processing steps of PCA dimensionality reduction, clustering, and visualization by UMAP were conducted. Cell identities were assigned by differential expression analysis and comparison to reported markers and/or as described in the text. We provide a table of the number of cells assigned to each cell type for our adult and juvenile thymus object and lymphoid/progenitor and myeloid marrow fractions object in Table S17. Additional subsetting on these objects was performed for subsequent analyses and standard processing procedures were applied.

Specifically, further subsetting was performed on the integrated lymphoid/progenitor and myeloid fractions processed with 39 principal components and clustered at a resolution of 0.8 to obtain the DC/NK development object (clusters 8, 21, 24, 28, 29, 32), B cell development object (clusters 3, 8, 19, 20, 21, 26, 28, 30), and T and NK cell objects (clusters 12, 24, 35). These subsetted objects were split and then re-integrated in the same manner as the full object. Note: clusters 8, 21, and 28 were progenitor clusters and were thus included in both the DC/NK and B cell development objects. Additionally, TECs and fibroblasts were subsetted from the adult and juvenile thymi processed with 50 principal components and a resolution of 1.5 by taking clusters 29, 31, 32, 39, and 42, splitting the object and re-integrating. We also subsetted, split, and re-integrated the T cell populations from the adult thymus object to perform T cell development trajectory analysis in Monocle 3. This subset excluded the non-T cell populations, such as B cells, TECs, DC-like cells (1) and (2), mixed, erythrocytes, NK-like cells, and macrophages. Cell type assignments were made similar to the full objects, and consistency of nomenclature was checked by extracting cell ids from the subsetted objects and confirming their location on the larger UMAP. All Seurat UMAPs of gene expression were generated with the FeaturePlot() function run on the RNA assay using default parameters except for the following specifications: cols = c(“lightgrey”, “red”), min.cutoff = 0, and order = TRUE. Genes visualized by dot plot were selected from among those identified through differential expression analysis using the FindAllMarkers() function in Seurat. In the majority of cases, we limited our results to genes expressed in a minimum of 25% of cells of a given population; but, in smaller datasets like our TEC, T, and NK-like cell, and B cell analyses, we reduced this minimum percentage to 15%. For the B cell analysis only when we also performed gene module analysis in Monocle 3 (see Gene module and trajectory analysis in Monocle 3), we included all genes highlighted from the gene module analysis individually for clarity, even if they did not meet these minimum cutoffs in Seurat (e.g., *pcdh7b*). For final inclusion, we considered the statistical significance, fold change, specificity, relation to known mammalian markers, and general interest. We include the full lists of differentially expressed genes for all dot plots generated in this fashion as supplemental datasets: Tables S1 (adult thymus), S2 (adult and juvenile thymus), S5 (TECs), S13 (T and NK-like cells), and S15 (B cells). Additionally, we provide a summary table of the lineage defining genes mentioned in our results section and how they relate to past work in zebrafish and known mammalian lineage markers in Table S18 (Agulleiro et al., 2010; Athanasiadis et al., 2017; Avagyan et al., 2021; Bachem et al., 2012; Bajoghli et al., 2009; Balla et al., 2010; Baracho et al., 2014; Baran-Gale et al., 2020; Baranov et al., 2016; Barbier et al., 2019; Baron et al., 2019; Barratt and Weitz, 2021; Bautista et al., 2021; Beetz et al., 2007; Ben-Moshe et al., 2010; Bhandari et al., 2016; Boller and Grosschedl, 2014; Bouwman et al., 2021; Brahler et al., 2018; Burda et al., 2010; Butko et al., 2015; Cadieux et al., 2005; Calvete, 1994; Carmona et al., 2017; Challen et al., 2014; Chan et al., 1997; Chen et al., 2021; Chong et al., 1995; Chopin et al., 2019; Dale and Topczewski, 2011; Danger et al., 2022; Danilova et al., 2005; De Decker et al., 2021; Dee et al., 2016; Di et al., 2017; Donovan et al., 2013; Ellmeier et al., 2013; Fallatah et al., 2022; Farahani and Xaymardan, 2015; Ferrero et al., 2020; Fu et al., 2009; Ganis et al., 2012; Garcia et al., 2018; Gioacchino et al., 2021; Gonzalez-Leal et al., 2014; Gordon et al., 2012; Gore et al., 2016; Hartmann et al., 2013; Hason et al., 2022; Haunerdinger et al., 2021; He et al., 2014; Hernandez et al., 2018; Hess and Boehm, 2012; Hey and O’Neill, 2016; Hildner et al., 2008; Hinton et al., 2004; Ho et al., 2009; Huang et al., 2020; Ikeda et al., 2017; Iwanami et al., 2011; Jeffrey et al., 2006; Jojic et al., 2013; Kadouri et al., 2020; Kalev-Zylinska et al., 2003; Karsunky et al., 2003; Kim et al., 2006; Kim et al., 2019; Klebanoff et al., 2013; Koenen et al., 2019; Koprunner et al., 2001; Kowalczyk et al., 2015; Kruse et al., 2009; Kuil et al., 2020; Lancaster et al., 2018; Langenau et al., 2004; Lee et al., 1994; Liao and Wang, 2021; Lieberman, 2010; Lieschke et al., 2002; Liu et al., 2015; Lloyd and Snelgrove, 2018; Lyons et al., 2001; Ma et al., 2012; Mahony et al., 2021*Preprint*; Marazuela et al., 2004; Martinez-Pomares, 2012; Matsumoto et al., 2008; Melichar et al., 2007; Mirabello et al., 2014; Mitchell et al., 2019; Modrell et al., 2017; Monney et al., 2002; Morath and Schamel, 2020; Murphy and Knee, 1994; Naganuma et al., 2011; Nemeth et al., 2003; Nitta et al., 2009; Nutt and Kee, 2007; Oettinger et al., 1990; Ohtani et al., 2008; Ong et al., 2020; Pandey et al., 2002; Parker et al., 2022; Parrish-Novak et al., 2000; Patel, 1999; Pearce et al., 2003; Perucho et al., 2014; Rajan et al., 2020; Remnant et al., 2021; Ridge et al., 2022; Rothenfusser et al., 2002; Rougeot et al., 2019; Ryu et al., 2005; Samborska et al., 2022; Schatz et al., 1989; Seeger et al., 1996; Shiau et al., 2015; Sichien et al., 2016; Sidney et al., 2014; Silacci et al., 2004; Smith et al., 1996; Stanley and Chitu, 2014; Sutoh et al., 2012; Takizawa et al., 2007; Tang et al., 2017; Trzpis et al., 2007; Tsai et al., 1994; Uechi et al., 2008; Ueno et al., 2004; Vanlandewijck et al., 2018; Venkateswarlu, 2003; Verlaet et al., 2002; Walton et al., 2015; Walzer et al., 2007; Wan et al., 2016; Wang et al., 2008; Wang and Peng, 2021; Wei et al., 2011; Wei et al., 2002; White et al., 2010; Willett et al., 1997a; Yeo et al., 2018; Ying et al., 2005; Yoder et al., 2002; Yoder et al., 2007; Yokota et al., 2003; Yoon et al., 2015; Zakrzewska et al., 2010; Zaslavsky et al., 2010; Zhang et al., 2013; Zhou et al., 2017; Zhu et al., 2012; Zimmerman et al., 2011). Our publicly available cell browsers also allow for the expression of any mapped gene of interest to be visualized (see Data availability).

R version 3.6.2 was used for all Seurat analyses. Monocle 3 analyses were carried out in R version 3.6.2 or 4.0.2.

### Comparing the composition of the adult and juvenile thymi

Cells of the adult and juvenile thymi were compared in two ways: (i) performing a two-group beta-binomial test of significance on a cell-type-by-cell-type basis using the countdata R package (Tables S3 and S6; Pham et al., 2010) and (ii) using the FindAllMarkers and FindMarkers functions in Seurat on the full object and within each cell type split by timepoint (adult vs. juvenile). Default parameters were used in all cases unless otherwise specified. Point (i) was applied to both the full adult and juvenile thymus in addition to the TEC/fibroblast subset. The full list of genes resulting from comparing adult vs. juvenile erythrocytes is provided in Table S4. To confirm that the ribosomal subunits identified as being differentially expressed in adult and juvenile thymi were not a technical artifact of inconsistent SoupX contamination removal, the uncorrected data was examined. Genes listed in the main text as being enriched in the adult thymus erythrocytes were likewise enriched in the uncorrected data.

### Clustering with RaceID3 and VarID for T cell trajectory analysis

For the analysis of T cell trajectories and the identification of ETPs, we performed an independent clustering of the adult thymi in RaceID3 (Herman et al., 2018). Following batch correction and the identification of outlier clusters, the RaceID3 SCseq object was subjected to integrated processing with VarID (Grun, 2020). More specifically, the RaceID3 SCseq object was generated from the raw data by calling the filterdata() function, with the parameters mintotal = 1,000, minexpr = 3, minnumber = 1, and the application of the internal RaceID batch correction method. This method identifies batch-associated genes through a local neighborhood approach and successive pair-wise merging (Herman et al., 2018). For this analysis, the size of the neighborhood, knn, was set to 10. Mitochondrial genes were regressed out for cell type inference, with ccor set to 0.15. Next, varRegression() was run to remove residual batch effects. Following these quality control steps, a distance matrix was computed and initial clusters determined by calling compdist() and clustexp() with default parameters. Outliers of the initial k-medoids clusters were identified with findoutliers(), a function that makes use of an internally computed background model of the expected gene expression variability. This model assumes a negative binomial distribution for transcript counts defined by the mean and variance of expression of each gene per cluster and has been validated for raw count data (Grun et al., 2014). Following the identification of outliers, rfcorrect() was run to improve the robustness of the final clustering. Next, clusters corresponding to cells in the T cell developmental pathway were subsetted for trajectory analysis; thus, clusters 4, 13, 14, 16, and 17 (corresponding to non-T cell populations, e.g., TECs, NK-like cells, B cells, etc.) do not appear in the UMAP shown in Fig. 2 A. VarID was used for subsequent processing on this subsetted UMAP. In brief, the function pruneKnn was run on the filtered expression matrix with large = TRUE, pcaComp = 100, regNB = TRUE, knn = 10, α = 1, and ngenes = 2,000, and links with probabilities lower than P value <0.01 were removed. The adjacency matrix was computed, Louvain clustering was performed, and the clusters were visualized. Gene expression was visualized by calling a color-scale modified version of the function fractDotPlot() with log_2_ transformation. FateID analysis was performed on this updated RaceID3 SCseq object. R version 4.1.0 was used for this analysis.

### Exploring T cell developmental trajectories with FateID

To investigate T cell trajectories, we applied the FateID algorithm to the RaceID3/VarID-generated object (Grun, 2020; Herman et al., 2018). In brief, FateID takes as input the user-defined most differentiated cells and implements an iterative random forest classification to calculate fate bias, working its way back to progenitor populations. Based on gene expression patterns consistent with mature populations, clusters 1, 19, and 5 were set as the endpoints in this analysis for trajectory 1 (mature T cells), trajectory 19 (cytotoxic T cells), and trajectory 5 (γδ T cells), respectively. In addition to specifying these endpoints, fatebias() was called using the following non-default parameters: the VarID distance matrix, minnr = 5, minnrh = 10, confidence = 0.85, and seed = 12345, and compdr() was used to visualize the dimensional reduction by UMAP. To observe the differentiation trajectories, principal curves were computed using the results from fateBias() and compdr() with a threshold of 0.15 for the fraction of random forest votes ascribed to a given trajectory. To investigate gene expression changes along pseudotime, cells with fate bias toward a given trajectory were extracted from the principal curve. Lowly expressed genes were removed by filtering the RaceID3-normalized transcript expression values using filterset() with minexp = 2 and minnumber = 1. A SOM of the pseudo-temporal order was generated using getsom() with the following inputs: the filtered gene set, nb = 100, and α = 0.5. This function groups genes with similar expression profiles into modules for further analysis. The SOM matrix was further processed with procsom() to group the initial modules into larger nodes, for which genes having >0.9 correlation of the SOM z-scores were combined into the same node. The minimal number of genes per node was set to 5. The processed SOMs were plotted with plotheatmap(). Table S7 lists the genes within each node. Pseudotime plots were generated using the FateID plotexpression() function for each gene in each trajectory, separately. Y axis upper limits were manually set to ensure these plots could be directly compared for each gene between trajectories 1 and 19; for some genes this upper limit excluded a small number of outlier cells for which inclusion in the plot would obscure the interpretation of finer expression level changes in pseudotime. α was set to 0.3 for all expression curves. R version 4.1.0 was used for the fate bias and pseudotime analysis of T cell development.

### Exploring B cell developmental trajectories with FateID

To investigate B cell developmental trajectories, we directly applied FateID to our B cell development Seurat object. Specifically, we stored the RNA count data and cell type classification as determined in Seurat and used these data as input to the fateBias() function with target clusters set to the mature IgD and IgT B cells and the following non-default parameters: minnr = 30, minnrh = 45, confidence = 0.95, and seed = 12345. Next, a dimensional reduction representation was generated using the compdr() function with m = c(“umap”) and umap$D2 was set using the cell embeddings from Seurat to preserve UMAP coordinates. Principal curves were computed using the results from fateBias() and compdr() and a threshold of 0.8 for the fraction of random forest votes ascribed to a given trajectory. This was purposefully set to be more stringent than in our T cell trajectory analysis due to the clearer assignment of B cells based on their immunoglobulin heavy chain gene expression. Investigations of gene expression changes in pseudotime were performed by calling the plotexpression() function for each gene of interest on the cells assigned to each trajectory independently. Y axis upper limits were manually set as above to allow for direct comparison of expression between trajectories and α values were set on a gene-by-gene basis to highlight expression changes in pseudotime. R version 4.1.0 was used for the fate bias and pseudotime analysis of B cell development.

### Identification of ETPs in thymus

To identify zebrafish ETPs, we employed multiple methods in addition to standard differential expression analysis which initially led to our use of this terminology. First, we integrated data from the adult thymus with that of the lymphoid/progenitor fraction of the adult marrow and highlighted the cell IDs of the cells from a high-resolution clustering of the adult thymus (resolution = 3) that were the strongest candidate ETPs (cluster 38). Clustering within the HSPC cluster of this integrated object would suggest that these cells were most similar transcriptionally to HSPCs in the marrow and offer additional support for this nomenclature. Next, we ensured the consistency between our ETP cell type calling within our Seurat and RaceID3 analyses and took advantage of our RaceID3 clustering to use StemID2 to infer the most progenitor-like population within the zebrafish thymus (Grun et al., 2016; Herman et al., 2018). The StemID2 score is computed based on the transcription entropy and number of inter-cluster links of each population, with less differentiated cell types tending to have greater transcription entropy and a higher number of inter-cluster links than more differentiated cell types (Grun et al., 2016; Herman et al., 2018). Transcriptional entropy was computed using default parameters; projections of cells onto inter-cluster links were computed with projcells() using default parameters except for setting nmode = FALSE. A P value threshold (pthr) of 0.01 was used as the cutoff for link significance. The StemID score, the product of the two values, was calculated for all clusters with compscore() and scthr = 0.8. Mean fate bias toward each trajectory was visualized for the clusters with the highest StemID scores (clusters 7, 11, 15, and 18). Error bars depict standard error of the mean (SEM). Additionally, for the two clusters with the highest StemID scores, we performed pathway analysis using Metascape (Zhou et al., 2019). Finally, we were able to take advantage of our kidney marrow analysis and previously reported markers of mouse and human ETPs in the literature to compute gene module scores in Seurat for three gene modules: (1) an HSPC module as determined by Seurat differential expression analysis (the 20 most specific HSPC genes (smallest pct.2 values) were assigned to this module), (2) an HSPC module as determined by Monocle 3 gene module analysis (module 16), and (3) an ETP module including orthologs of highly specific mouse and human ETP markers as determined by literature review (Table S9; Cante-Barrett et al., 2017; Cordes et al., 2022*Preprint*; Porritt et al., 2004; Rothenberg et al., 2008; Zeng et al., 2019). In most cases, all zebrafish orthologs were included in this gene module with the exception of *gata2a*, previously shown to mark eosinophils (Balla et al., 2010), and *cd44c* due its lack of inclusion in the current mapping.

Once the ETPs were identified in the combined adult lymphoid/progenitor marrow fraction and thymus object, the Seurat FindMarkers() and FindConservedMarkers() functions were called to determine differentially expressed genes between the thymic ETPs and marrow HSPCs (cluster 4 in object visualized with 35 PCs and resolution = 0.6) and also the conserved genes between these populations (Tables S10 and S11). All cells within cluster 4 were used for the purpose of assessing conserved markers and the markers of highest conservation are reported here, defined by expression in <5% of non-cluster 4 cells derived from the thymus and <25% of non-cluster 4 cells derived from the marrow. A more stringent cutoff was used for the thymus due to the relatively sparse recovery of most transcripts (low pct.1 values for specific markers); however, we note that this did eliminate genes like *si:ch211-161c3.6*, which were less specific but more sensitive, in this case recovered in 94% of thymic ETPs but also 15% of other thymic cells. Our criteria also favored the recovery of myeloid lineage genes due to the lower stringency in the marrow. This approach was advantageous from the point of view of identifying highly specific ETP markers that were also expressed in HSPCs. For other analyses and goals, different filtering criteria may be desired. Upon noting a broader population of thymic cells included in this cluster beyond our putative ETPs, for differential expression analysis, only the most specific subset of thymic ETPs from the adult thymus object (cluster 38, resolution = 3) were compared to cluster 4 marrow cells. Significantly enriched genes in HSPCs or ETPs had to meet the following criteria: be detected in >40% of the population, have a ln fold change >0.4 over the other tissue’s population, and be detected in <5% of the other tissue’s population. Wishing to minimize the recovery of differentially expressed genes simply due to tissue differences, FindMarkers() with a min.pct of 0.02 was additionally called on the B cells of the marrow vs. the B cells of the thymus (cells within clusters 2 and 19). Genes that met the criteria above but were identified as being differentially expressed in thymic vs. marrow B cells and were recovered in <10% of marrow B cells were not included in the final gene lists for HSPC or ETP enrichment. None of the marrow HSPC enriched genes were eliminated from this analysis; however *lck*, *runx3*, *FP236356.1*, *ccr9b*, and *bcl11ba* were removed from the ETP enrichment list. While a true enrichment in any of these genes may be present, this elimination allows us to be more conservative in our analysis. We note that although *pdpk1a* was also identified as a differentially expressed gene in marrow vs. thymic B cells, we have chosen to include it in our enrichment list because its expression in thymic ETPs is less likely to be due to tissue-specific transcript contamination as detection in marrow B cells was non-negligible at 14.4% (vs. 30.5% in thymus). Eosinophilic markers were excluded from the set of markers enriched in kidney marrow HSPCs because it was observed that they were present only in a specific subset of eosinophil progenitors.

### Gene module and trajectory analysis in Monocle 3

The developer branch of Monocle 3 was used in all analyses (Cao et al., 2019). Seurat objects were first converted to Monocle 3 cell_data_set objects using the as.cell_data_set() function, thus preserving the dimensionality reduction, clustering, and cell annotation from Seurat. The nearest neighbor graph was rebuilt in Monocle 3 and used to conduct trajectory analysis and compute pseudotime. Standard functions were employed for differential gene expression analysis.

To enable plotting in Monocle 3, the gene_name and gene_short_name metadata slots were populated manually via calling the rownames() and rowData() functions for the desired inputs and estimate_size_factors() was run.

For each of the following objects, the following deviations from the default parameters were used to define clusters and learn trajectory graphs: full marrow object: resolution 0.003; DC/NK object: resolution = 0.009, ncenter = 1,300; B cell object: resolution = 0.009, ncenter = 400, minimal_branch_len = 15; thymus T cell subset: resolution = 0.004, ncenter = 1,200. (Note: Trajectory analysis was not performed on the T and NK cell object, a subset of the full marrow object, so the Seurat clustering was used in downstream Monocle 3 analysis.)

Following clustering and trajectory determination, differential gene expression analysis was conducted using the graph_test function, which performs spatial autocorrelation analysis using the Moran’s I test. As recommended, the principal_graph was selected as the neighbor graph to use in this analysis and identifies genes that vary over the computed trajectory instead of between clusters. To find modules of co-regulated genes, find_gene_modules() was called on a subset of the original object only containing differentially expressed genes with q_values set to <0.05 and the resolutions listed above (Tables S8, S12, and S16). Standard processing was followed for visualizing the computed gene modules and exploring module specificity. Verification of individual genes identified as being specific to populations of interest was performed in Seurat.

The most differentially expressed genes between the T and NK cell populations were identified through calling the top_markers() function grouping the cells by lineage, as assigned from the full marrow object (Table S14).

For B cell pseudotime analysis, all cells from the B cell and progenitor object in addition to 11 genes of interest: *pax5*, *cd79a*, *sid1*, *dntt*, *rag1*, *rag2*, *csf1rb*, *ebf3a*, *mki67*, *igl3v5*, and *igl1c3* were subsetted. An ordering of the cells was determined by interactively selecting the root state to be within the HSPC population from within the order_cells() function. The pseudotime ordering was then observed for all genes of interest with the genes ordered according to time of expression and a minimum untransformed expression level of 0.1. Note: *ebf3a*, although not ascribed as the direct ortholog of the B cell commitment factor *EBF1* in mammals, appeared to have the most consistent expression with *EBF1* and was thus selected as a B cell gene. Further, we note that although the IgT and IgD populations are two independent lineages, we did not separate them on the pseudotime plot for the purposes of exploring early B cell development when they appeared intermixed. The genes we selected for subsetting would not be anticipated to be expressed differently between these two lineages.

For T cell pseudotime analysis, all cells from the subsetted adult thymus object containing only T cell populations along with the following seven genes of interest: *cd4-1*, *cd4-2.1*, *cd4-2.2*, *cd8a*, *cd8b*, *csf1rb*, and *il2rb* were subsetted. Based on the expression patterns of the *CD4* and *CD8* orthologs, cells were selected using the choose_cells() function for pathway analysis of the mature T cells and cytotoxic T cells, respectively. These two subsets were ordered using the same root selected from within the ETP population. Next, we modified the plot_genes_in_pseudotime() function to visualize single cells expressing orthologs of *CD4* and *CD8* simultaneously. Specifically, we plot the sum of all *CD4* orthologs, the sum of all *CD8* orthologs, and the minimum of those sums as the joint CD4-CD8 expression using a minimum untransformed expression level of 0.1.

### TCR alignment and analysis

TCR α and β constant regions were not annotated in zebrafish Ensembl GRCz11 (the closest annotation is FP236356.1, a 109,805 bp region that includes TCR β constant 1 [*trbc1*]). Therefore, an independent alignment was performed to recover TCR α and TCR β transcripts based on the recovery of their constant regions, the most 3′. A custom genome was defined with three genes, two individually for the four Cb1 (TCRb.C1) and three Cb2 exons (TCRb.C2), respectively (2), and one for the TCR α constant region (TCRa.C) as annotated in GenBank listing AF424545.1. The custom alignment was processed through Cell Ranger, similarly to the full transcriptome mapping, and assigned as “threeprime” chemistry. The filtered_feature_bc_matrix files were loaded into Seurat in a way that preserved all of the cellular barcodes as identified by Cell Ranger (CreateSeuratObject settings min.cells = −1, min.features = −1). All datasets from which more than one cell was recovered were merged, and the cell ids corresponding to counts of TCRa.C > 0, TCRb.C1 > 1, and TCRb.C2 > 0 were retrieved. αβ T cells were defined as meeting any of the above criteria. Similarly, cell ids corresponding to counts of *trdc* > 0 and *tcrg* (*si:dkeyp-13d12.23*) > 1 were also retrieved from the original transcriptome mappings. γδ T cells were defined as meeting either or both of those requirements for *trdc* and *tcrg* recovery. These transcript cutoffs were set based on the observed sensitivity and specificity of cutoff choice in the marrow and used consistently in the thymus. We note that this choice appears less specific in the thymus but we maintained the marrow cutoffs for sensitivity, consistency, and transparency.

### Online supplemental material

Fig. S1 shows an extended cell type characterization of the adult zebrafish thymus in addition to supporting characterizations of the adult and juvenile thymus, including visualization of cells by timepoint, an investigation into the mixed granulocyte population, and ribosomal subunit heterogeneity. Fig. S2 shows supporting visualizations and analysis for the TEC and fibroblast subset of the adult and juvenile thymus along with supplemental visualizations of the FateID analysis and identification of zebrafish ETPs. Fig. S3 shows conserved marker genes between marrow HSPCs and ETPs. Fig. S4 shows differentially expressed genes between marrow HSPCs and ETPs. Fig. S5 shows representative plots of the fluorescence-activated cell sorting gates for marrows and thymi. Table S1 shows differentially expressed genes in the adult zebrafish thymus. Table S2 shows differentially expressed genes from the adult and juvenile zebrafish thymi. Table S3 shows beta-binomial test results for the adult and juvenile zebrafish thymi. Table S4 shows differentially expressed genes in adult and juvenile erythrocytes from the adult and juvenile thymi analysis. Table S5 shows differentially expressed genes in the TEC and fibroblast subset of the adult and juvenile thymi. Table S6 shows beta-binomial test results for the TEC and fibroblast subset from the adult and juvenile thymi. Table S7 shows gene lists for SOM nodes from the FateID analysis of the adult thymus. Table S8 shows Monocle 3 gene modules of the adult zebrafish kidney marrow. Table S9 shows gene modules used to identify zebrafish ETPs. Table S10 shows conserved marker genes between marrow HSPCs and ETPs. Table S11 shows differentially expressed genes between marrow HSPCs and ETPs. Table S12 shows Monocle 3 gene modules of the DC-like cell and NK-like cell marrow subset. Table S13 shows differentially expressed genes for marrow T and NK-like cell subset. Table S14 shows top distinguishing genes between marrow T and NK-like cells. Table S15 shows differentially expressed genes for marrow B cell subset. Table S16 shows Monocle 3 gene modules of the marrow B cell subset. Table S17 shows cell counts by cell type for full adult and juvenile thymi and marrow analyses. Table S18 shows marker gene references.

## Supplementary Material

Table S1 shows differentially expressed genes for adult zebrafish thymus.

Table S2 shows differentially expressed genes for adult and juvenile zebrafish thymi.

Table S3 shows beta-binomial test results for adult and juvenile zebrafish thymi.

Table S4 shows differentially expressed genes in adult and juvenile erythrocytes from thymus analysis.

Table S5 shows differentially expressed genes for TEC and fibroblast subset of adult and juvenile thymi.

Table S6 shows beta-binomial test results for TEC and fibroblast subset of adult and juvenile thymi.

Table S7 shows gene lists for SOM nodes in FateID (adult thymus).

Table S8 shows Monocle 3 gene modules for adult zebrafish kidney marrow.

Table S9 shows gene modules used to identify zebrafish ETPs.

Table S10 shows conserved marker genes between marrow HSPCs and ETPs.

Table S11 shows differentially expressed genes between marrow HSPCs and ETPs.

Table S12 shows Monocle 3 gene modules for marrow DC-like and NK-like cell subset.

Table S13 shows differentially expressed genes for marrow T and NK-like cell subset.

Table S14 shows top distinguishing genes between marrow T and NK-like cells.

Table S15 shows differentially expressed genes for marrow B cell subset.

Table S16 shows Monocle 3 gene modules for marrow B cell subset.

Table S17 shows cell counts by cell type for full adult and juvenile thymi and marrow analyses.

Table S18 shows marker gene references.

## Data Availability

The data reported in this article have been deposited in the Gene Expression Omnibus database under accession no. GSE190794. Additionally, cell browser objects (Speir et al., 2021) have been generated for ease of data exploration and are available at the following URL: https://dr-marrow-thymus.cells.ucsc.edu.
